# Time-Resolved Probing of the Iodobenzene C-Band Using XUV-Induced Electron Transfer Dynamics

**DOI:** 10.1021/acsphyschemau.4c00036

**Published:** 2024-08-10

**Authors:** James Unwin, Weronika O. Razmus, Felix Allum, James R. Harries, Yoshiaki Kumagai, Kiyonobu Nagaya, Mathew Britton, Mark Brouard, Philip Bucksbaum, Mizuho Fushitani, Ian Gabalski, Tatsuo Gejo, Paul Hockett, Andrew J. Howard, Hiroshi Iwayama, Edwin Kukk, Chow-shing Lam, Joseph McManus, Russell S. Minns, Akinobu Niozu, Sekito Nishimuro, Johannes Niskanen, Shigeki Owada, James D. Pickering, Daniel Rolles, James Somper, Kiyoshi Ueda, Shin-ichi Wada, Tiffany Walmsley, Joanne L. Woodhouse, Ruaridh Forbes, Michael Burt, Emily M. Warne

**Affiliations:** † Chemistry Research Laboratory, Department of Chemistry, University of Oxford, Oxford OX1 3TA, United Kingdom; ‡ School of Chemistry, University of Southampton, Highfield, Southampton SO17 1BJ, United Kingdom; § Linac Coherent Light Source, SLAC National Accelerator Laboratory, 2575 Sand Hill Road, Menlo Park, California 94025, United States; ∥ PULSE Institute, SLAC National Accelerator Laboratory, 2575 Sand Hill Road, Menlo Park, California 94025, United States; ⊥ National Institutes for Quantum Science and Technology (QST), SPring-8, 1-1-1 Kouto, Sayo, Hyogo 679-5148, Japan; # Department of Applied Physics, Tokyo University of Agriculture and Technology, Tokyo 184-8588, Japan; ∇ Department of Physics, Kyoto University, Kyoto 606-8502, Japan; ○ Department of Chemistry, Graduate School of Science, Nagoya University, Nagoya, Aichi 464-8602, Japan; ◆ Department of Applied Physics, Stanford University, Stanford, California 94305-4090, United States; ¶ Graduate School of Material Science, University of Hyogo, Kouto 3-2-1, Kamigori-cho, Ako-gun, Hyogo 678-1297, Japan; ⋈ National Research Council of Canada, 100 Sussex Dr. Ottawa, ON K1A 0R6, Canada; ⧓ Institute for Molecular Science, Okazaki 444-8585, Japan; ⧖ Department of Physics and Astronomy, University of Turku, Turku FI-20014, Finland; ● Graduate School of Advanced Science and Engineering, Hiroshima University, Higashi-Hiroshima 739-8526, Japan; ¤ Department of Chemistry, School of Science, Tokyo Institute of Technology, 2-12-1 W4-10 Ookayama, Meguro-ku, Tokyo 152-8551, Japan; ☼ Japan Synchrotron Radiation Research Institute, Kouto 1-1-1 Sayo, Hyogo 679-5198, Japan; ◎ RIKEN SPring-8 Center, Kouto 1-1-1 Sayo, Hyogo 679-5148, Japan; ◑ School of Chemistry, George Porter Building, University of Leicester, Leicester LE1 7RH, United Kingdom; ◐ J. R. Macdonald Laboratory, Department of Physics, Kansas State University, Manhattan, Kansas 66506, United States; ◯ Department of Chemistry, Tohoku University, Sendai 980-8578, Japan

**Keywords:** femtochemistry, photochemistry, molecular dynamics, free electron laser spectroscopy, extreme ultraviolet spectroscopy, site-selective ionization, electron transfer dynamics

## Abstract

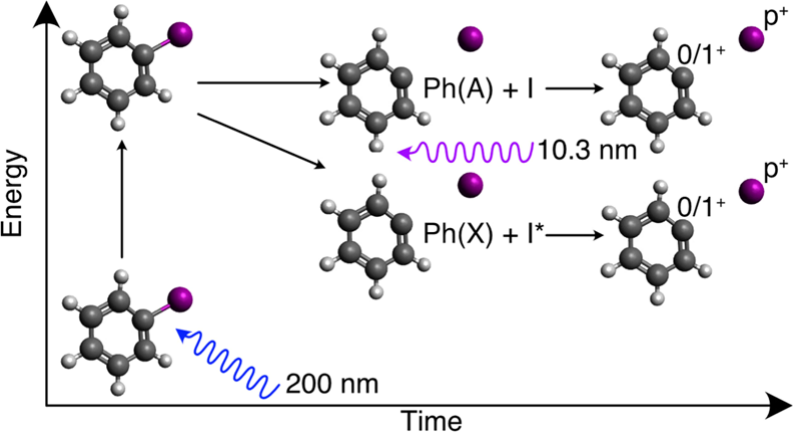

Time-resolved extreme ultraviolet spectroscopy was used to investigate photodissociation within the iodobenzene C-band. The carbon–iodine bond of iodobenzene was photolyzed at 200 nm, and the ensuing dynamics were probed at 10.3 nm (120 eV) over a 4 ps range. Two product channels were observed and subsequently isolated by using a global fitting method. Their onset times and energetics were assigned to distinct electron transfer dynamics initiated following site-selective ionization of the iodine photoproducts, enabling the electronic states of the phenyl fragments to be identified using a classical over-the-barrier model for electron transfer. In combination with previous theoretical work, this allowed the corresponding neutral photochemistry to be assigned to (1) dissociation via the 7B_2_, 8A_2_, and 8B_1_ states to give ground-state phenyl, Ph(X), and spin–orbit excited iodine and (2) dissociation through the 7A_1_ and 8B_2_ states to give excited-state phenyl, Ph(A), and ground-state iodine. The branching ratio was determined to be 87 ± 4% Ph(X) and 13 ± 4% Ph(A). Similarly, the corresponding amount of energy deposited into the internal phenyl modes in these channels was determined to be 44 ± 10 and 65 ± 21%, respectively, and upper bounds to the channel rise times were found to be 114 ± 6 and 310 ± 60 fs.

## 1 Introduction

Iodobenzene photochemistry has received significant attention at a variety of excitation wavelengths from 193 to 350 nm, in part due to the complex dynamics arising from the dual chromophore of the phenyl ring and iodine atom.^1−13^ Time-resolved studies have predominantly focused on exciting iodobenzene within the A- and B-bands of its absorption spectrum, which span roughly 205–350 nm. At these wavelengths, rapid carbon–iodine photolysis is thought to occur due to nσ* transitions akin to those observed in alkyl iodides.^2,5,7,10,12,14,15^ Relatively slower dissociation mechanisms have additionally been attributed to large quantities of energy being partitioned into the vibrational modes of the phenyl ring through ππ* transitions, followed by predissociation to nσ* states and loss of iodine.^2,7,10^ For both of these reaction pathways, signals corresponding to the production of I(^2^P_3/2_) and I(^2^P_1/2_) (hereafter referred to as I and I*) have been observed.^5−9,13^

An extensive nanosecond resonance-enhanced multiphoton ionization (REMPI) velocity map imaging (VMI) study by Sage et al. has previously examined the origins of the I and I* products over the 206–330 nm range, covering the majority of the A-band and reaching the lower limit of the B-band.^13^ The principle transitions contributing to the A-band were determined to have πσ* and nσ* character, with the former corresponding to excitation to the 2A_1_ state and loss of I and the latter to excitation to the 4A_1_ state and production of I*. By contrast, the photochemistry of the B-band was shown to be dominated by a ππ* excitation to the 5A_1_ state, which primarily results in I*. However, these are just the major contributors, and it is worth noting that at least four I channels and three I* channels were distinguishable in the REMPI measurements. Their corresponding product angular distributions exhibited anisotropic and isotropic recoil with decreasing anisotropy at higher photon energies. This is consistent with the fast and slow dissociation mechanisms outlined above and, in the latter case, with contributions from multiple electronic states of different symmetries.

**Figure 1:**
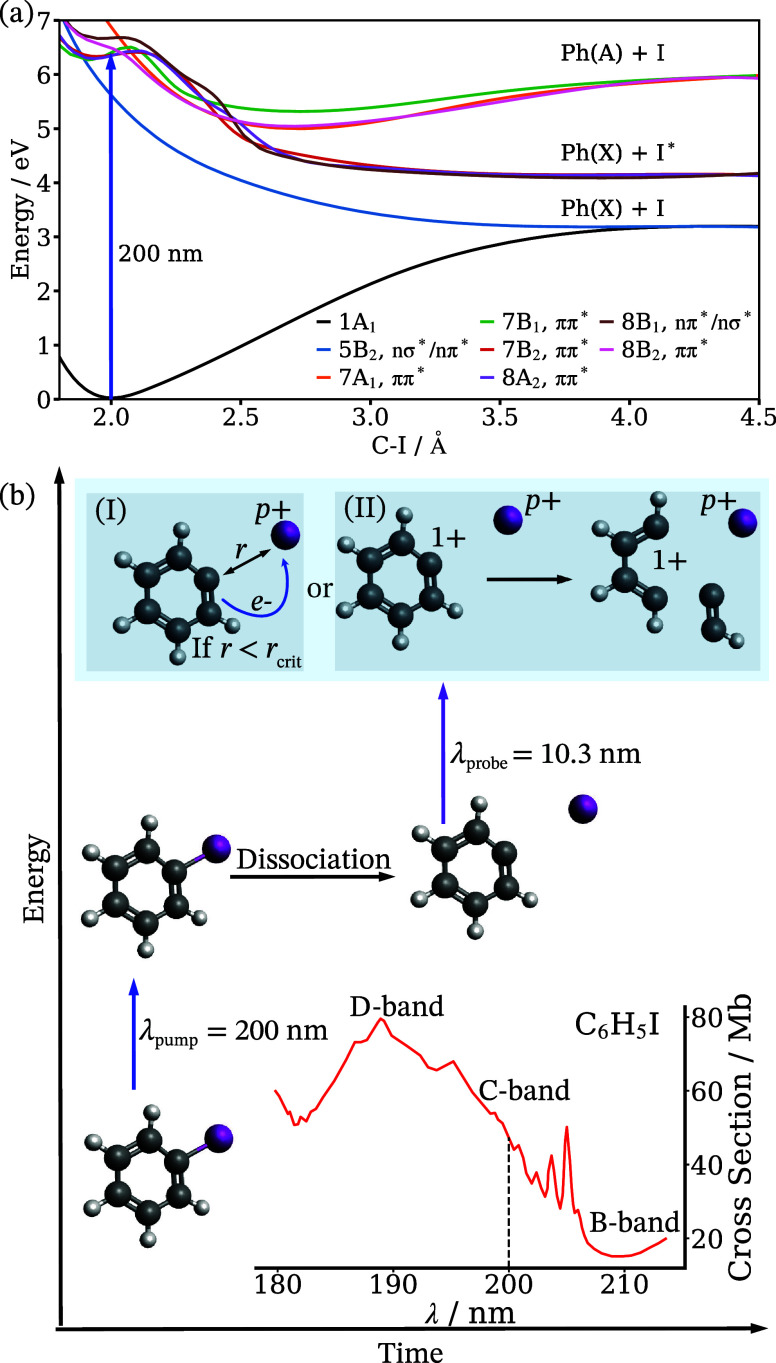
(a) Calculated potential energy curves along the C–I bond coordinate for the relevant states discussed in this report (adapted from ref (16), copyright 2016 ACS Publications). (b) Schematic depicting iodobenzene photolysis at 200 nm and subsequent probing of the neutral fragments at 10.3 nm. The pump pulse can initiate two competing C–I dissociation mechanisms, one rapid and one slow (see the main text), resulting in different fragment momenta that depend on the internal excitation of the cofragments. The probe pulse, which is tuned to selectively ionize the iodine atom, enables these channels to be differentiated using two pump–probe signatures. First, the iodine can initially be ionized to a *p*+ charge state while the phenyl fragment remains neutral. Electron transfer can then occur if the fragments are nearby, producing Ph^+^ and I^(*p*–1)+^ (I). Second, the probe pulse can directly produce Ph^+^ with I^*p*+^, resulting in a Coulomb explosion (II). In this scenario, Ph^+^ can subsequently break into multiple fragments. A representative fragmentation pathway is shown here, but multiple outcomes are possible. The UV-absorption spectrum for iodobenzene from the edge of the B-band, which is centered at 227 nm, and into the C-band, which is centered at 200 nm, is also shown, with the pump wavelength indicated by a dashed line.^1,29^

Significantly less attention has been paid to wavelengths that lie within the iodobenzene C-band, centered at 200 nm.^1^ Much of our understanding comes from a femtosecond time-resolved ion yield study by Hu et al. at 200 nm, which was supported by CASPT2 calculations of spin–orbit resolved potential energy curves along the C–I coordinate.^16^ They found that in contrast to the A- and B-bands, three dissociation channels are accessible at 200 nm, with only two likely to proceed. Their calculations demonstrated that this wavelength allows the population of the 7B_1_ and 7B_2_ states through nπ* and ππ* transitions that are inaccessible in the B-band. These can lead to three outcomes, which are illustrated in Figure 1a.^13,16^ First, the 7B_1_ and 7B_2_ states overlap with the 5B_2_ state, which in principle could produce ground-state phenyl, Ph(X), and I. However, migrating to this state involves a slow transition from the triplet 7B_1_ and 7B_2_ states to the predominantly singlet 5B_2_ state, and as such, no evidence for this outcome was found. Instead, the experimental observations were assigned to rapid transitions from the 7B_1_ and 7B_2_ states to the 7A_1_ and 8B_2_ states. From these points, the excited molecule can eventually dissociate to give I and electronically excited Ph(A), or it can further migrate to any of the 7B_2_, 8A_2_, or 8B_1_ states, producing Ph(X) and I*. However, due to the signal-to-noise ratio of these measurements, no branching ratio for Ph(X) and Ph(A) could be determined. These experiments were also insensitive to the formation of I*, as the authors specifically probed an I(^2^P_3/2_) resonance.

In this report, time-resolved extreme ultraviolet (XUV) spectroscopy is used to gauge the Ph(X) and Ph(A) branching ratio in the iodobenzene C-band and to explore the corresponding photodissociation dynamics. This approach is a reliable tool for distinguishing such properties as well as reaction lifetimes and molecular geometry changes through transient states.^17−27^ Here, we present a 200 nm pump/10.3 nm probe study of the photodissociation of iodobenzene. The ultraviolet (UV) pump pulse initially cleaves the C–I bond, while the probe pulses are tuned above the iodine 4d edge to enable site-selective resonant absorption and multiple ionization of the liberated iodine atom (I or I*) through Auger–Meitner decay.^28^ By analyzing the time-dependent features that arise from the resulting charged iodine fragments and their interactions with neutral or ionic phenyl fragments, this report identifies and assigns the two possible iodobenzene dissociation mechanisms proposed by Hu et al. at 200 nm.^16^ These time-dependent features are shown schematically in Figure 1b, which illustrates that the pump–probe features derive from interfragment electron transfer from a neutral phenyl radical to an iodine cation (channel I) as well as from time-dependent Coulomb explosions between two ionic cofragments (channel II).^17^ To our knowledge, this report is the first example of interfragment electron transfer, induced by site-selective ionization, being used as a probe for assigning the electronic state of a neutral photoproduct.

## 2 Experimental Section

Pump–probe spectroscopy measurements were made at the soft X-ray beamline (BL1) of the SPring-8 Angstrom Compact free electron LAser (SACLA), using the velocity-map ion imaging spectrometer developed by Ueda and co-workers.^30^ The configurations of the optical and free electron lasers at SACLA BL1 have been detailed previously and are only outlined here.^31,32^ Briefly, pump pulses [λ_pump_ = 200 nm, bandwidth = 1.1 nm (fwhm)] were generated with durations of less than 200 fs from the fourth harmonic of a 10 mJ, 40 fs, 800 nm beam. The 800 nm pulses were produced using a mode-locked oscillator (Vitara, Coherent Inc.), a chirped pulse amplification system (Legend Elite, Coherent Inc.), and a custom-built multipass amplifier. The UV pulse energy and focal spot diameter in the interaction region were 1 μJ and 88 μm, respectively, which provided a UV intensity of 1.6–3.2 × 10^11^ W cm^–2^. The probe pulses produced by SACLA [λ_probe, XUV_ = 10.3 nm, bandwidth = 0.2 nm (fwhm)] originated from a bunched electron beam that was accelerated into a series of undulators to produce XUV light with an average pulse energy of 11.4 ± 1.1 μJ.^31^ The full XUV energy distribution is shown in Figure S1 of the Supporting Information. This was reduced to 1.33 ± 0.13 μJ in the interaction region due to attenuation by a 0.65 μm Zr filter and additional losses along the beamline. The probe intensity was therefore estimated to be 1.1 × 10^14^ W cm^–2^, using a focal spot size of 10 μm and a pulse duration of 30 fs.^33^

The delay between the two laser pulses was controlled by using an automated delay stage, and the measurements were recorded in 50 fs steps. Pulse arrival times were then further refined to approximately 10 fs accuracy using a jitter-correcting timing tool.^34^ The time when the two pulses arrived simultaneously in the interaction region, *t*_0_, was determined by looking at the onset times of the Coulomb curves in the resulting ion momentum distributions. The assignment of *t*_0_ is expanded upon in the Supporting Information. Gaseous iodobenzene was introduced into the interaction region using a pulsed supersonic molecular beam that was crossed at an angle of 45° by the pump and probe pulses. Ions generated from these pulses were directed through a time-of-flight tube by electrostatic optics with velocity mapping potentials and were amplified and recorded by two microchannel plates coupled to a hexanode delay-line detector.^30,33,35,36^ This yielded positional and temporal (*x*, *y*, and *t*) values for each event, allowing the momentum and mass-to-charge ratios (*m*/*z*) of each ion hit to be determined.

Subsequent to the experiments at SACLA BL1, supporting pump–probe measurements were made at Femtolab Oxford using an in-house velocity-map imaging spectrometer and a Ti:sapphire laser system (Solstice Ace, Spectra-Physics). In these measurements, the XUV-probe pulses were replaced by 47 fs, 240 μJ infrared (IR) pulses [λ_probe, IR_ = 800 nm, bandwidth = 20 nm (fwhm)], which were focused to roughly 60 μm in the laser interaction region of the spectrometer to yield an average intensity of 3.3 × 10^14^ W cm^–2^. The pump pulses were provided by frequency-converting a portion of the 800 nm beam (TOPAS Prime, Light Conversion) to 200 nm, yielding 100 fs and 7 μJ pulses. The maximum UV intensity was therefore 4.1 × 10^12^ W cm^–2^, approximately the same as the SACLA experiment, taking a focal spot size of 64 μm. Ions were detected using a dual microchannel plate and a P47 detector, and the resulting photons were subsequently imaged by a Pixel Imaging Mass Spectrometry (PImMS) camera equipped with a PImMS2 sensor.^37^

## 3 Results and Discussion

**Figure 2:**
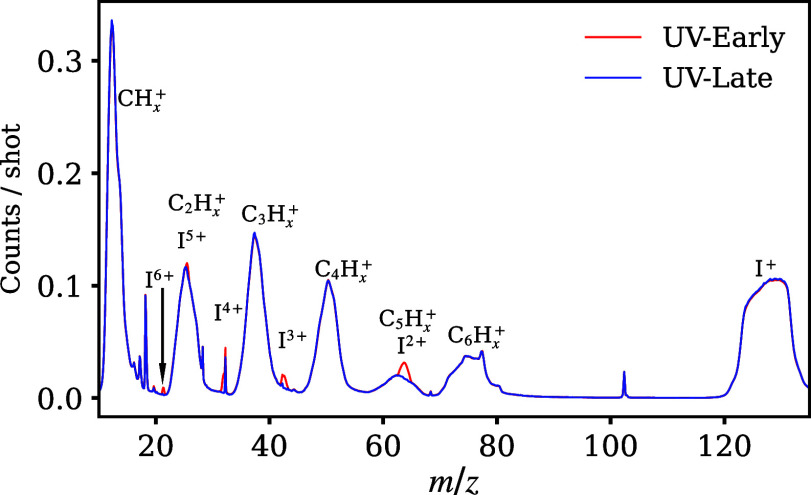
Iodobenzene mass spectra for the pump–probe delays where UV pulses precede (red) or follow (blue) the XUV interaction. Iodine charge states from I^1–6+^ were observed as well as those from C_*x*_H_*y*_^+^ fragments with *x* = 1–6 and *y* = 1–5. UV-early corresponds to the UV pulse arriving prior to the XUV pulse, and UV-late corresponds to the UV pulse arriving after the XUV pulse. Time-dependent enhancements are seen in the I^2–6+^ fragments when the UV pulse precedes the XUV.

Multiple ionic fragments can be produced by the UV-pump and XUV-probe pulses. When iodobenzene interacts solely with the XUV pulse, I^*p*+^ states with *p* = 1–6 are produced along with singly charged C_*x*_H_*y*_ ions (with *x* ranging from 1 to 6 and *y* from 1 to 5). This distribution of charge states arises because the I, C, and H atoms have absorption cross sections of approximately 1.4 Mb, 0.1 Mb, and 0.01 Mb, respectively, at 10.3 nm, creating a strong preference for ionization of the iodine atom.^38,39^ When the UV pulse precedes the XUV interaction (“UV-early”), additional time-dependent features are visible in the iodine signals where *p* = 2–6. No time dependence is seen in any of the carbon fragments. Figure 2 shows time-of-flight mass spectra for the UV-early and UV-late interactions with iodobenzene, where enhancements in the multiply charged iodine signals are visible. Here, “UV-late” corresponds to the XUV pulse arriving before the UV. The UV-early spectrum therefore includes signals from molecules that have been excited prior to being probed, while the UV-late spectrum corresponds to probing of the ground-state molecule by the XUV (and to a lesser extent XUV-pump/UV-probe interactions). The UV-late and UV-early delay ranges include all data before or after *t*_0_ and are normalized to their respective numbers of laser shots. Ion images of the UV-early enhancements are provided in Figure S2 of the Supporting Information and exhibit isotropic fragment angular distributions, suggesting contributions from multiple electronic states of different symmetries or relatively long reaction lifetimes. This is also consistent with the measurements of Sage et al., where decreasing anisotropy was seen as the wavelength was shortened from 250 to 206 nm.^13^

**Figure 3:**
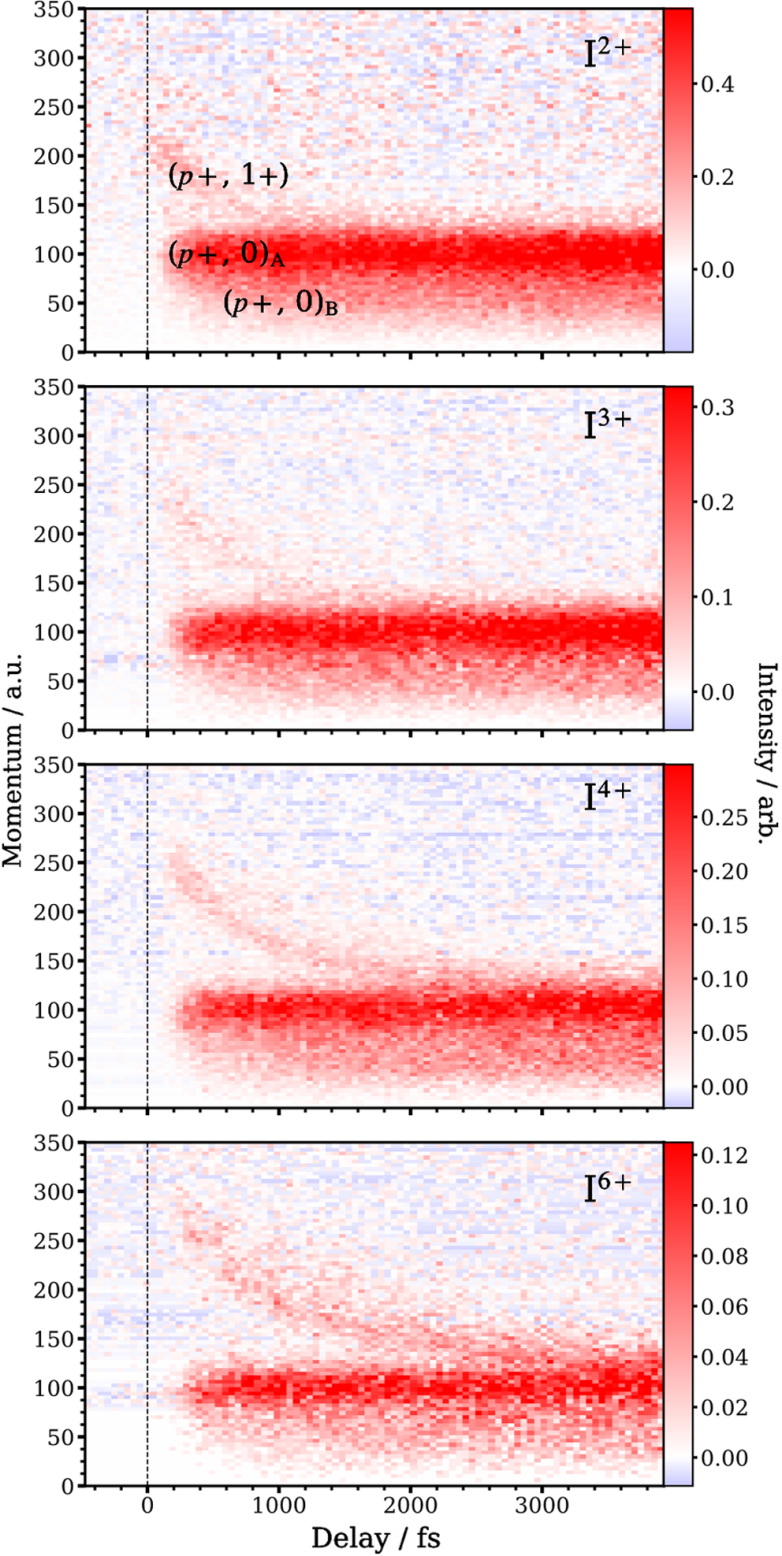
Delay-dependent 3D momentum distributions for I^2,3,4,6+^. The distributions were produced by removing the component where the XUV pulse arrives prior to the UV pulse (obtained by averaging data from −500 to −100 fs prior to *t*_0_). Three enhancements are visible in all iodine charge states and include a time-dependent Coulomb curve, corresponding to I^*p*+^ recoiling against another charged fragment (*p*+, 1+), as well as two low, constant velocity channels, (*p*+, 0)_A_ and (*p*+, 0)_B_ that correspond to I^*p*+^ being produced with a neutral cofragment. Momenta are expressed in atomic units.

Delay-dependent three-dimensional (3D) momentum distributions of I^2,3,4,6+^ are shown in Figure 3 over a −0.5 to 3.9 ps range. These exhibit the time-resolved momentum information for each ion and only show contributions from the probing of UV-excited molecules, as any signal from the ground state has been removed by subtracting averaged data from a −500 to −100 fs range before *t*_0_. The unsubtracted momentum distributions are shown in Figure S3 of the Supporting Information. The I^5+^ measurements were excluded from this analysis due to their significant *m*/*z* overlap with that of C_2_H_*y*_^+^. Figure 3 exhibits three distinct enhancements for each ion, which are described using (*p*+, *q*+) notation, where *p*+ corresponds to the iodine charge state and *q*+ can be 0 or 1+, corresponding to the charge on the cofragment. The two features with low, constant momentum observed for each iodine charge state are assigned as (*p*+, 0) channels (Figure 1b, channel I), where the charged iodine recoils against one or more neutral cofragments. These appear in the momentum distributions as sharp features centered at roughly 100 atomic units (au, defined as ℏ/*a*_0_), which appear promptly after *t*_0_, and also as delayed features centered around 50 au. The presence of two independent low momentum channels with different onset times suggests the existence of two reaction pathways, consistent with the work of Hu et al. at 200 nm.^16^ The third feature is a Coulomb curve, which is the result of two or more neutral fragments becoming charged by the XUV pulse and recoiling against each other (Figure 1b, channel II).^17,21,26,40,41^ These curves, which start at high momenta and gradually decay with increasing delay, are assigned as (*p*+, 1+) channels. These correspond to a charged iodine recoiling against a singly charged cofragment. The following analysis confirms the assignments of the (*p*+, 0) and (*p*+, 1+) channels and uses them to extract information about the underlying photochemistry of iodobenzene.

### 3.1 (*p*+, 0) Dissociation

When the iodine ions recoil against neutral fragments, they accelerate to a distribution of constant velocities purely by gaining translational energy from any remaining photon energy after neutral C–I dissociation except that which is partitioned into the phenyl ring. For iodobenzene, these low momentum channels can therefore be used to determine several photochemical properties: the identities and electronic states of the neutral cofragments following dissociation; the amount of internal energy deposited into the phenyl ring; the branching ratio of the two observed dissociation channels.

**Figure 4:**
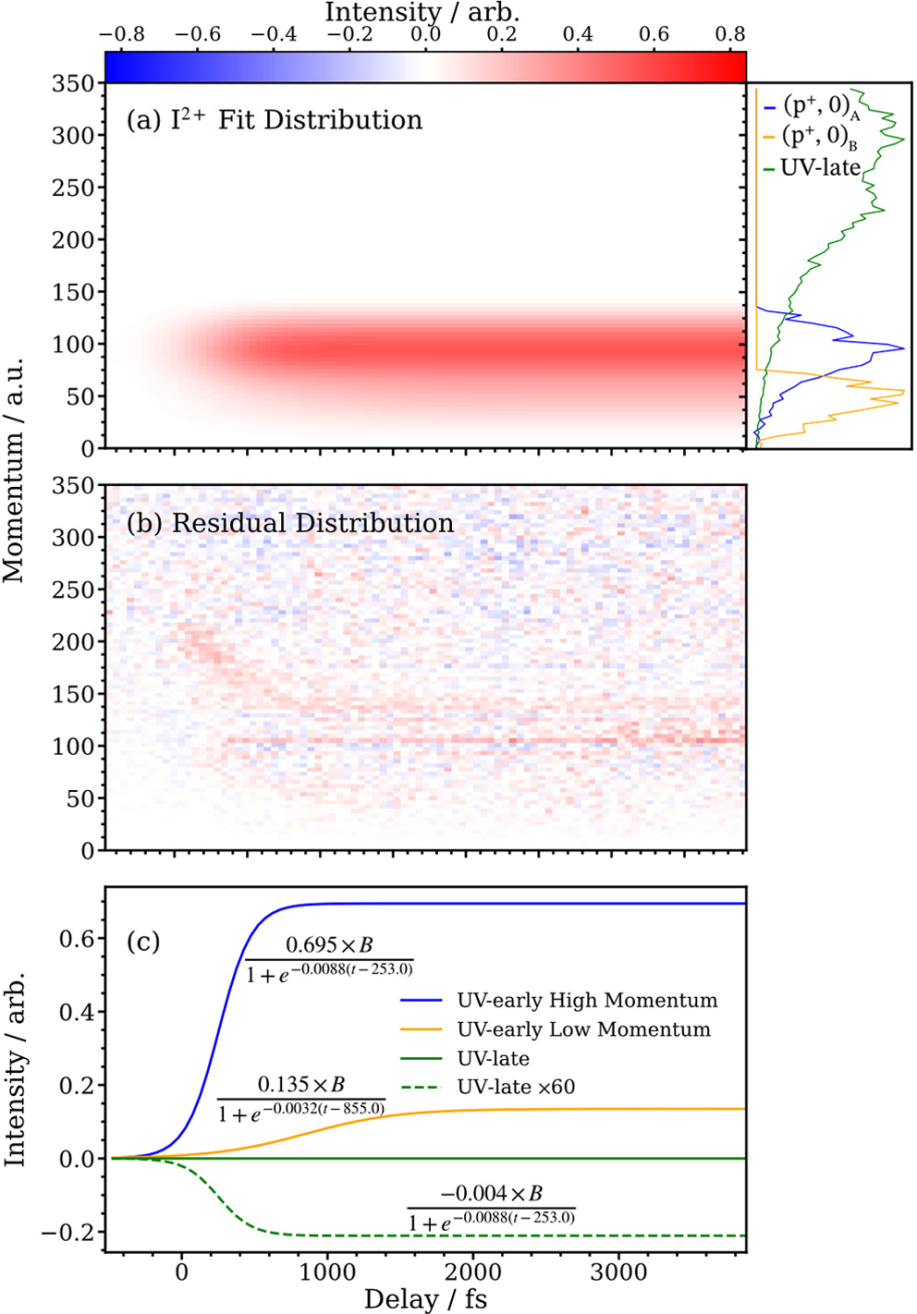
Basis function reconstruction of the I^2+^ pump–probe data, created by fitting eq 1 using a least-squares approach. Panel (a) shows the summed contributions of the momentum-dependent basis functions with respect to the pump-probe delay. The side panel additionally exhibits the selected basis functions used for the global fit, normalized to their maximum intensities. Panel (b) demonstrates the residual data created when the optimized fit is subtracted from the experimental data. Panel (c) shows the individual contribution of each basis function to the reconstructed data along with the optimized parameters determined from the fitting procedure.

To determine the above information, the (*p*+, 0)_A_ and (*p*+, 0)_B_ channels observed in Figure 3 were initially isolated using the adapted global fitting procedure described by Razmus et al.^27^ As expected, two independent basis functions were identified as the principal contributors to the momentum ranges of these channels (approximately 0–150 au) in the time-dependent I^2–4+^ momentum distributions, with a third providing a significantly smaller contribution to the I^2+^ data. These are shown in Figure 4a for I^2+^ and in Figure S4 of the Supporting Information for I^3+^ and I^4+^. The I^6+^ signal was not analyzed further here due to the low intensity of the signal and its overlap with other low *m*/*z* fragments. The I^2–4+^ basis functions correspond to channels (*p*+, 0)_A_ and (*p*+, 0)_B_, and the additional component for I^2+^ represents the depletion of a signal that originates from the fragmentation of ground-state parent molecules by the XUV pulse. For I^2+^, the basis function for the ground-state depletion, which occurs over a broad momentum range, was obtained from the UV-late signal, where the XUV pulses interact with unperturbed molecules, from −500 and −200 fs; the (*p*+, 0)_A_ basis function was determined by averaging the data after *t*_0_ and before the onset of the (*p*+, 0)_B_ channel, between 75 and 425 fs; the (*p*+, 0)_B_ basis function was then itself determined by subtracting the (*p*+, 0)_A_ basis function from the overlapping momentum distributions at later delays, 3 to 4 ps, as well as the remaining contribution from the (*p*+, 1+) channel, which overlaps with the (*p*+, 0) features at long delays (this latter subtraction is detailed in the Supporting Information). Reconstructed momentum distributions were then obtained by least-squares fitting the I^2–4+^ momentum distributions to the summed logistics functions shown in eq 1 (where *p* represents momentum rather than charge number). Each logistic function represents one of the processes described above and uses the corresponding basis function as an input.
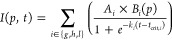
1

In eq 1, the three channels (denoted by *g*, *h*, and *l*, respectively, for ground-state depletion as well as high- and low-velocity enhancement) are characterized by *A*, the amplitude of the pertinent basis function; *B*(*p*), the momentum-dependent basis function itself; *k*, the phenomenological rate constant for the signal rise or depletion; *t*, the pump–probe delay; *t*_crit_, the onset times of the photoinduced channels, which are determined from the centers of their signal rises. With these parameters, the summed intensity *I*(*p*,*t*) of these channels is calculated as a function of momentum and time. Figure 4 illustrates this reconstructed momentum distribution for I^2+^ as well as the optimized parameters for each logistic function and their corresponding contributions at each delay point. The residual of the global fit is also shown and essentially isolates and reproduces the (*p*+, 1+) feature observed in Figure 3. Analogous figures for I^3–4+^ are shown in Figures S5 and S6 of the Supporting Information.

**Figure 5:**
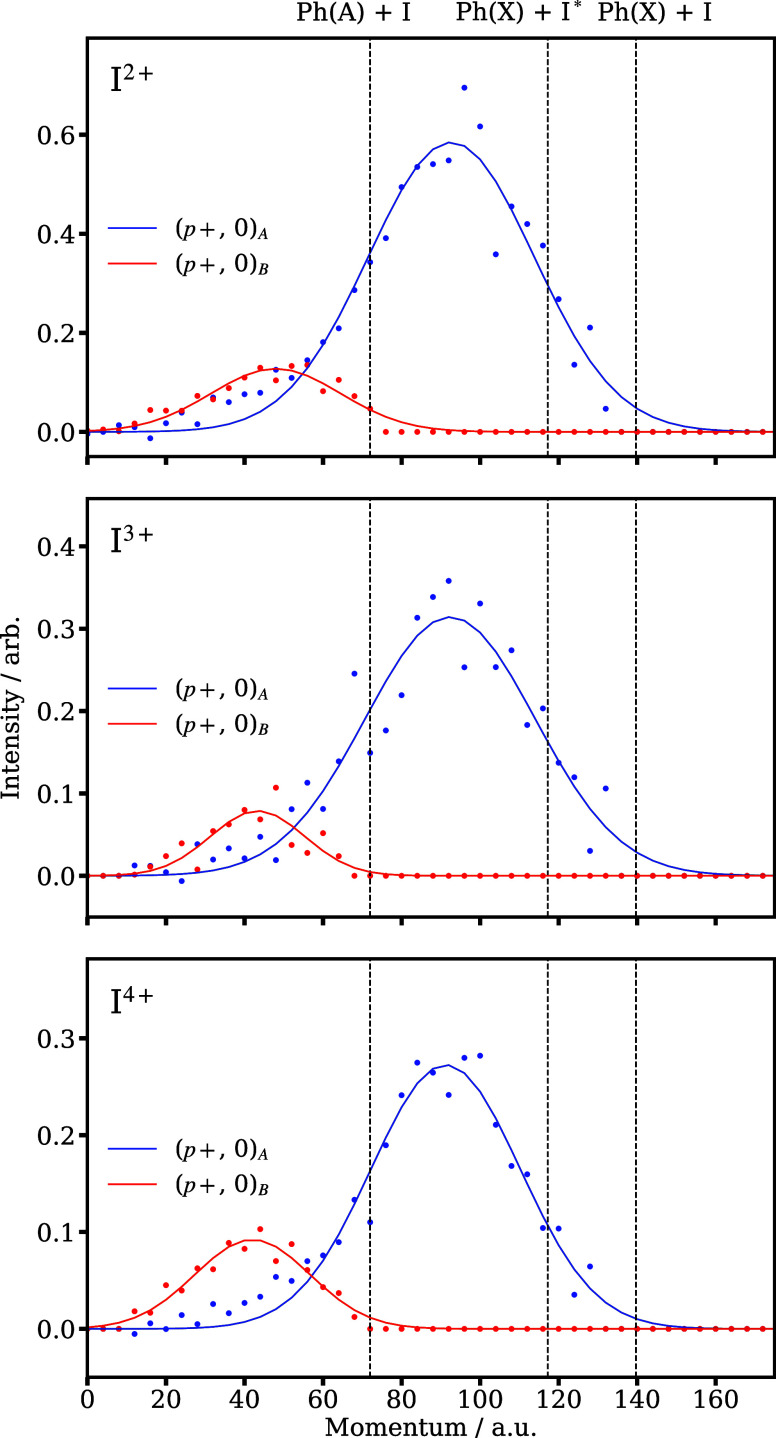
Momentum distributions obtained for the (*p*+, 0) basis functions from the global fitting technique for I^2–4+^, which isolates the overlapping channels observed in Figure 3. The channel intensities are scaled by the amplitudes of the optimized logistics functions. The dashed lines represent the maximum possible momentum of each iodine ion when no energy is partitioned into the internal modes of the phenyl ring for the three likely dissociation channels.^16^

Figure 5 illustrates the momentum distributions of channels (*p*+, 0)_A_ and (*p*+, 0)_B_ for the I^2–4+^ data. These were obtained from the selected basis functions and scaled using the amplitude parameters shown in Figure 4c. Averaging the Gaussian centers and standard deviations of the (*p*+, 0)_A_ and (*p*+, 0)_B_ channels across the I^2–4+^ charge states yields neutral iodine velocities of 870 ± 190 and 420 ± 130 ms^–1^, respectively, which correspond to total fragment kinetic energy releases of 1.32 ± 0.29 and 0.31 ± 0.10 eV, assuming C–I cleavage. These product kinetic energies are significantly lower than the energy available following dissociation through any of the three reaction pathways proposed by Hu et al. (Figure 5, dashed lines), indicating significant excitation of the phenyl moiety.^16^

The fit to eq 1 that produces the reconstructed momentum distributions also returns parameters corresponding to the onset times and rate constants of the (*p*+, 0) channels. These are given in Table 1. The onset times of the (*p*+, 0) channels can be used to determine the excited states of the phenyl cofragments involved in each mechanism. As mentioned previously, these delayed appearances are indicative of electron transfer processes.^17,20,21,24,26^ In this case, after iodobenzene dissociates to produce I or I*, the liberated iodine becomes positively charged after the probe pulse arrives. At short times after *t*_0_, the I^*p*+^ charge states can then be reduced by electron transfer from the neutral phenyl, allowing the two product fragments to coexist as I^(*p*–1)+^ and Ph^+^, assuming no subsequent dissociation of the latter. When this process occurs, the two charged fragments repel, resulting in significantly higher fragment momenta than that gained from the neutral dissociation alone. The onset times of the low-velocity channels in the I^*p*+^ momentum distributions therefore represent the upper limits of this time range and are the points at which the separations of the two fragments are great enough that electron transfer can no longer occur. In principle, electron transfer signatures should also be observable in the corresponding I^(*p*–1)+^ momentum distributions as Coulomb curves that exist over short pump–probe delays. However, as these features have high momenta they often overlap energetically with the fragments originating from direct Coulomb explosions of the parent molecule and are often difficult to isolate.^20^

**Table 1:** Optimized Onset Times (*t*_crit_) and Rate Constants (*k*) Determined for Each Iodine Charge State Using the Global Fitting Method for the (*p*+, 0) Channels

ion	*t*_crit,A_ (fs)	*t*_crit,B_ (fs)	*k*_A_ (×10^–3^ fs^–1^)	*k*_B_ (×10^–3^ fs^–1^)
I^2+^	253 ± 7	850 ± 70	8.8 ± 0.5	3.2 ± 0.6
I^3+^	294 ± 6	870 ± 50	8.4 ± 0.4	3.2 ± 0.5
I^4+^	333 ± 7	1100 ± 50	8.0 ± 0.4	2.4 ± 0.3

The *t*_crit_ values obtained from the global fit, given in Table 1 as *t*_crit,A_ and *t*_crit,B_, correspond to the onset times of the (*p*+, 0) features. It is worth noting that these onset times increase with the iodine charge state as higher charge state ions lengthen the distance over which electron transfer remains viable (see eq 2, below). To confirm the effectiveness of the global fitting procedure, these onset times are compared to those obtained directly from the experimental data by fitting cumulative distribution functions (CDFs). The results are provided in Table 2, and the CDF fits are provided in Figure S7 of the Supporting Information. These were carried out using the 70–125 au momentum ranges in Figure 3, which correspond to the (*p*+, 0)_A_ channels. CDF fitting has previously been used in electron transfer studies to determine the onset times of isolated channels, and so the strong agreement seen between the *t*_crit_ values of the (*p*+, 0)_A_ channels obtained from the global fit, and those obtained from directly fitting CDFs to the (*p*+, 0)_A_ channels, confirms the capability of the global fit to characterize the overlapping channels observed here.^20,25,26,41^ It was not possible to obtain an accurate value of *t*_crit_ for the (*p*+, 0)_B_ channels via CDF fitting, as their delayed onsets and relative intensities mean there is no region where they do not overlap with the (*p*+, 0)_A_ channels, and so their appearance times are always influenced by the latter.

**Table 2:** Experimental and Calculated Over-the-Barrier (OTB) Model Values for *t*_crit_, the Time at which Electron Transfer Can No Longer Occur for Each Iodine Charge State^a^

(*p*+, 0)_A_				
I^*p*+^	GF exp. *t*_crit_ (fs)	CDF exp. *t*_crit_ (fs)	OTB *t*_crit_ (fs) Ph(X)	OTB *t*_crit_ (fs) Ph(A)
I^2+^	250 ± 50	240 ± 50	290 ± 60	410 ± 90
I^3+^	290 ± 50	280 ± 50	340 ± 70	470 ± 110
I^4+^	330 ± 50	320 ± 50	380 ± 80	530 ± 120

(*p*+, 0)_B_				
I^*p*+^	GF exp. *t*_crit_ (fs)		OTB *t*_crit_ (fs) Ph(X)	OTB *t*_crit_ (fs) Ph(A)
I^2+^	850 ± 90		600 ± 190	850 ± 270
I^3+^	870 ± 70		700 ± 220	990 ± 310
I^4+^	1100 ± 70		780 ± 250	1100 ± 350

a Experimental values were determined using the onset times of the observed (*p*+, 0) channels, either directly by cumulative distribution function (CDF) fitting or through the global fitting (GF) approach involving eq 1, as described in the main text. A 50 fs error was propagated into the experimental uncertainties to account for the precision of the *t*_0_ assignment. Over-the-barrier onset times were calculated using the *r*_crit_ values determined by eq 2, which were subsequently converted to *t*_crit_ values by using the experimental fragment velocities, which are the source of the reported standard deviations. Values are provided for the (*p*+, 0)_A_ and (*p*+, 0)_B_ channels, with the cofragment assumed to be either Ph(X) or Ph(A).

To identify the electronic state of the cofragment, the *t*_crit_ values are compared to those predicted by the over-the-barrier (OTB) model described by eq 2.^42^ This model predicts the internuclear separation, *r*_crit_, at which the electron transfer can no longer occur, which can then be converted to a channel onset time using the corresponding fragment velocities.
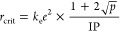
2

In eq 2, *e* is the charge on an electron, *k*_e_ is the Coulomb constant, *p* is the charge number of the iodine, and IP is the ionization potential of the neutral cofragment from which the electron is migrating. After the C–I dissociation, there is not enough available energy following the UV excitation for the phenyl ring to undergo any subsequent dissociation.^43^ Considering this, the probable candidate for the cofragment is a neutral phenyl radical. The *t*_crit_ values for the two (*p*+, 0) channels were compared to those predicted for an OTB charge transfer from a neutral phenyl radical (Table 2), using different ionization potentials to account for the previously reported Ph(X) and Ph(A) states.^16^ Ionization potentials of 8.32 and 5.89 eV were used for the Ph(X) and Ph(A) states, respectively.^44,45^ The experimental and OTB *t*_crit_ values reported in Table 2 strongly agree when a Ph(X) cofragment is assumed for the (*p*+, 0)_A_ channel and a Ph(A) fragment for the (*p*+, 0)_B_ channel. The equivalence between these two sets of values demonstrates the sensitivity of the electron transfer channels to the electronic state of the cofragment and confirms the channels originate from the production of ground-state phenyl and spin–orbit excited iodine (Ph(X) + I*) and excited phenyl with ground-state iodine (Ph(A) + I). Potential contributions from I produced with Ph(X) are disregarded here due to its spin-forbidden nature and lack of prior observation at 200 nm.^16^

The global fitting procedure also yielded phenomenological rate constants for each (*p*+, 0) channel, *k*_A_ and *k*_B_, which include contributions from the temporal resolution of the experiment, the decay rates to yield Ph(X) + I* or Ph(A) + I, and the interfragment distance-dependency of the electron transfer probability in each channel. This latter contribution can lead to slower rise times with increasing charge state and hence deviations from the behavior predicted by the over-the-barrier model. This has been demonstrated for electron transfer reactions between methyl radicals and I^4–14+^ following the dissociation of CH_3_I.^17^ This same trend is observed in the I^2–4+^ data reported here, with *k*_A_ decreasing from (8.8 ± 0.5)× 10^–3^ fs^–1^ to (8.0 ± 0.4)× 10^–3^ fs^–1^ with increasing charge state and *k*_B_ decreasing from (3.2 ± 0.6)× 10^–3^ fs^–1^ to (2.4 ± 0.3)× 10^–3^ fs^–1^. As such, of the I^*p*+^ rate constants reported here, the I^2+^ results are likely to exhibit the smallest deviation from the classical over-the-barrier model. They can therefore be used to provide upper bounds of 114 ± 6 and 310 ± 60 fs to the rise times (1/*k*) of the Ph(X) and Ph(A) fragments.

The lifetimes reported here are broadly in accordance with the convoluted phenyl rise time (combined for both the Ph(X) and Ph(A) channels) of 290 ± 90 fs measured by Hu et al. using a Ph^+^ transient signal.^16^ However, it is worth pointing out that they additionally reported an I^+^ transient rise time of 1.2 ± 0.2 ps, which they assigned to the overall rate of reaction, beginning with the excitation of the phenyl modes and resulting in the production of ground-state iodine. The discrepancy between the Ph^+^ and I^+^ time scales has not been fully characterized, but the agreement between the rise time upper limits reported here and their Ph^+^ measurements may suggest that this arises from the choice of probe wavelength and the differing origins of the I^+^ signal. The I^+^ transient yield measured by Hu et al. may contain contributions produced from dissociative ionization of the parent molecule, in addition to signal from 298.2 nm (2 + 1) REMPI of free iodine. By contrast, the (*p*+, 0) channels reported here arise primarily from site-selective core ionization of an iodine 4d electron (and subsequent Auger decay) after C–I photolysis.

The amount of energy partitioned into the phenyl ring can also be determined for each channel using conservation of energy arguments.^40,41^ Starting with the (*p*+, 0)_A_ channel, assigned here to the production of Ph(X) and I*, this can be determined by applying the known spin–orbit excited iodine dissociation energy of 3.85 eV^13^ to
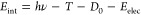
3where *E*_int_ is the internal energy of the phenyl ring, *h*ν is the energy of the 200 nm photon (6.20 eV), *T* is the translational kinetic energy of the two fragments, *D*_0_ is the dissociation energy, and *E*_elec_ is the relative energy of the phenyl electronic state (0 and 2.43 eV for Ph(X) and Ph(A), respectively).^45^ A similar process can be applied to the (*p*+, 0)_B_ channel to determine the amount of energy partitioned into internal modes of the Ph(A) fragment, using the ground-state iodine dissociation energy, 2.9 eV.^13^ The results of both of these calculations are provided in Table 3, where it can be seen that, for the (*p*+, 0)_A_ dissociation, 44 ± 10% of the available energy following C–I cleavage is deposited into the phenyl ring and that this proportion grows to 65 ± 21% for the (*p*+, 0)_B_ channel.

**Table 3:** Internal Energy of the Phenyl Ring after C–I Dissociation at 200 nm^a^

channel	I^*p*+^ (eV)	*T* (eV)	*E*_int_ (eV)	*f*_int_ (%)
(*p*+, 0)_A_	0.50 ± 0.11	1.32 ± 0.29	1.03 ± 0.23	44 ± 10
(*p*+, 0)_B_	0.12 ± 0.04	0.31 ± 0.10	0.57 ± 0.18	65 ± 21

a I^*p*+^ is the average energy of the iodine fragment across all charge states determined from the total kinetic energy released. *T* is the total translational kinetic energy of the two fragments, *E*_int_ is the energy distributed into the phenyl ring, and *f*_int_ is the fraction of available energy partitioned into internal excitation of the phenyl ring expressed as a percentage.

Finally, the intensities of each of the low momentum channels obtained from the optimized global fit can also be used to determine the branching ratio of the dissociation routes, assuming that the 4d ionization cross sections of I and I* are roughly the same. Here, this is accomplished using the amplitudes and areas of the (*p*+, 0)_A_ and (*p*+, 0)_B_ basis functions (as shown in Figures 4 and 5). The branching ratios for the I^2–4+^ data, as well as the average across all charge states, are shown in Table 4. Here, it can be seen that 87 ± 4% of the molecules dissociate via the high-momentum (*p*+, 0)_A_ channel into Ph(X) and I*, while the remaining 13 ± 4% follow the low momentum channel to produce Ph(A) and I.

**Table 4:** Branching Ratios of the (*p*+, 0)_A_ and (*p*+, 0)_B_ Channels, Representing Dissociation into Ph(X) + I* and Ph(A) + I, Respectively^a^

ion	(*p*+, 0)_A_ (%)	(*p*+, 0)_B_ (%)
I^2+^	87 ± 2	13 ± 2
I^3+^	89 ± 6	11 ± 6
I^4+^	83 ± 15	17 ± 15
average	87 ± 4	13 ± 4

a The mean branching ratios and standard deviations are weighted values determined by using the relative intensities of the I^*p*+^ channels.

### 3.2 (*p*+, 1+) Dissociation

The (*p*+, 0) channels demonstrate that C–I photolysis at 200 nm produces neutral Ph(X) and Ph(A) cofragments. It is therefore curious that although strong (*p*+, 1+) features (Coulomb curves) are evident for each iodine charge state, they do not appear in the phenyl momentum distribution or for any other carbon fragment (see Figure S8 of the Supporting Information). These would be expected, as a charged cofragment must at some point exist for the iodine (*p*+, 1+) channels to occur. Moreover, no time dependence was seen in any covariance analysis between ion pairs. This lack of time dependence means that there is no direct confirmation of the charged cofragment that leads to the (*p*+, 1+) channels. Even so, the lack of available energy for further phenyl fragmentation after C–I bond cleavage, as suggested by the decomposition energetics modeled by Lin and co-workers, combined with the excellent agreement between the observed and predicted (*p*+, 0) channel onset times, strongly indicates that the phenyl radical is initially intact before it is ionized by the 10.3 nm pulse.^43^

As will be demonstrated below, analysis of the Coulomb curves provides further confirmation that the phenyl radical is the neutral cofragment by establishing that the (*p*+, 1+) channel involves a phenyl monocation. These (*p*+, 1+) curves represent two or more ions recoiling against each other after the interaction with the XUV pulse. Since the electrostatic component of this repulsion decreases with increasing separation (and hence increasing pump–probe delay), at long delays the observed velocities of the iodine fragments will approach the value expected following neutral C–I dissociation. Using these asymptotic velocities, the appearance of the Coulomb curves for different masses and cofragment charge states can be modeled. This was performed here by taking the final neutral dissociation velocities of the iodine ions from the (*p*+, 0) channels and determining their separation velocities with respect to potential neutral photofragments (Ph, C_3_H_3_, and C_2_H_2_) using conservation of momentum arguments. The results were then used to determine the expected distances between iodine and the putative cofragments immediately after dissociation and up to a chosen time. Coulomb’s law was then used to introduce a Coulombic component to these velocities, to account for the additional velocity gained from electrostatic repulsion.^21,26,40^ In this study, two simulated Coulomb curves are produced for each potential fragment combination, as iodobenzene can dissociate through the Ph(X) + I* or Ph(A) + I pathways. The appearances of these curves differ depending on the charge and momentum of the chosen cofragment, allowing their presence or absence to be determined by a comparison with the experimental data.

**Figure 6:**
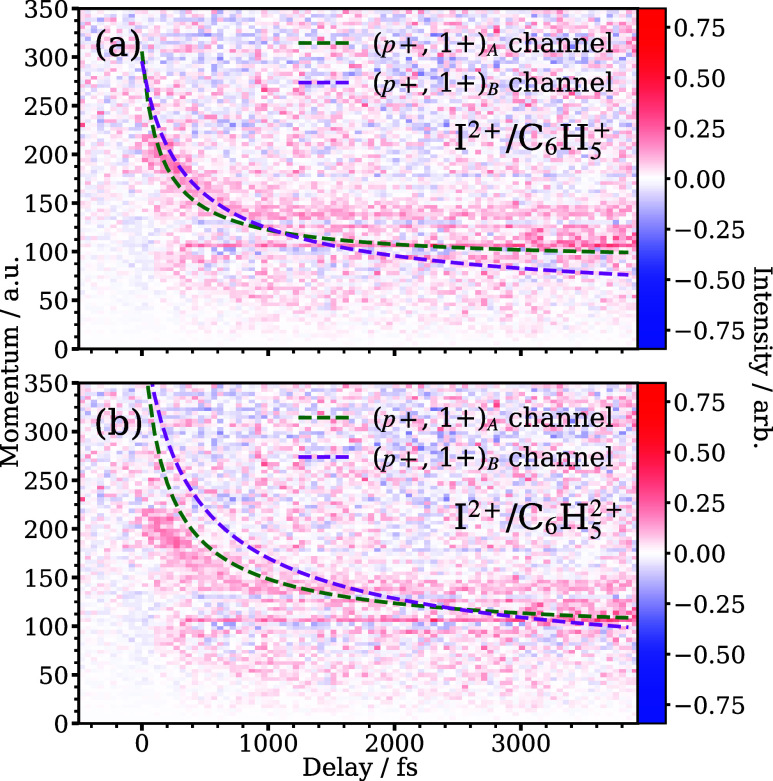
Panel (a): Simulated Coulomb curves for the two-body explosion of I^2+^ against Ph^+^ at different pump–probe delays, compared with the residual (*p*+, 1+) curve isolated by the global fitting procedure. The (*p*+, 1+)_A_ curve was modeled using the fragment velocities produced via the higher momentum dissociation, while the (*p*+, 1+)_B_ curve was produced using the lower momentum dissociation. Two ionization outcomes are shown here: (a) I^2+^ and C_6_H_5_^+^ and (b) I^2+^ and C_6_H_5_^2+^. The positive charge on the phenyl fragment is placed on the site of C–I cleavage.

Figure 6a illustrates the modeled curves for I^2+^ recoiling against Ph^+^ for both the Ph(X) + I* (green) and Ph(A) + I (purple) dissociation channels. The simulations both match the residual (*p*+, 1+) curve isolated by the global fitting procedure, and their similarity also explains why distinct curves are not observed for each channel. The experimental and modeled curves for the higher iodine charge states also exhibit strong agreement when using Ph^+^ as a cofragment (Figure S9 of the Supporting Information). Attempts at modeling the (*p*+, 1+) channel using cofragments with different *m*/*z* were not able to reproduce the experimental data, with the output demonstrating differences in curve shape, origin, and asymptotic velocity. An example of this is shown in Figure 6b, where the modeled curves were produced with a phenyl dication cofragment. Additional examples using smaller carbon cofragments are shown in Figure S10 in the Supporting Information and also exhibit poor agreement with the experimental data. It should be noted that, when modeling the phenyl cation, the charge was placed on the carbon of the ring at the site of C–I cleavage. Figure S11 of the Supporting Information demonstrates that shifting the charge further away from the site of C–I cleavage does not significantly change the agreement with the experimental measurements.

The combination of the modeled Coulomb curves from the (*p*+, 1+) explosion and the strong agreement of the (*p*+, 0)_A_ OTB calculations from the previous section provide indirect confirmation that the phenyl radical is the cofragment after C–I bond cleavage, even though no delay-dependent properties were observed in any of the carbon-containing ion momentum distributions. To directly validate this assignment, additional measurements were recorded using the 200 nm UV/800 nm IR pump–probe experiment described previously. Figure S12 in the Supporting Information shows the time-dependent UV/IR momentum distributions produced from this experiment, and it can be seen that in this system a time-dependent Coulomb curve was observed in the phenyl fragment. Subsequent time-dependent recoil-frame covariance analysis of this data (Figure S13 in the Supporting Information) confirmed that C_6_H_5_^+^ is produced in the same reaction channel as I^+^, as would be expected for the mechanisms assigned in this report.^46−50^ The nonobservation of time-dependent Ph^+^ after iodobenzene interacts with both the UV and XUV pulses is therefore attributed to its fragmentation prior to reaching the detector, which does not occur when a different ionization regime is applied by the IR pulse.^51^ This highlights a potential complication of soft X-ray or XUV-probe methods: they are not purely site-selective and can potentially place cofragments in a variety of excited electronic states that lead to fragmentation. In this case, the absorption of a 120 eV photon enables the phenyl moiety to fragment in multiple ways, producing various hydrocarbon fragments as well as atomic and molecular hydrogen.^52^ As a consequence, any time-dependent signal associated with the phenyl may be diffused across multiple carbon-containing channels or remain undetected as high-momentum H^+^ or H_2_^+^ fragments.

## 4 Conclusions

The iodobenzene C-band was investigated at 200 nm by using time-resolved XUV spectroscopy. Two neutral product channels were isolated from the (*p*+, 0) iodine pump–probe features using a global fitting method.^27^ Their onset times, intensities, and momenta enabled them to be assigned to the production of 87 ± 4% Ph(X) + I* and 13 ± 4% Ph(A) + I, with 44 ± 10 and 65 ± 21% of the available energy following C–I dissociation being deposited into the internal phenyl modes, respectively. In this analysis, the phenyl electronic states were confirmed by comparing the channel onset times with those predicted using an over-the-barrier model for electron transfer. To our knowledge, this is the first demonstration of electron transfer processes initiated by site-selective ionization being used to distinguish the electronic states of initially neutral cofragments.

By comparing the above results with prior theoretical work by Hu et al., it can be concluded that the higher momentum (*p*+, 0)_A_ channel likely arises from initial excitation of iodobenzene to the 7B_1_ and 7B_2_ states, followed by internal conversion to the 7A_1_ and 8B_2_ states and subsequent (re)crossing to the 7B_2_, 8A_2_, and 8B_1_ manifold and dissociation into Ph(X) and I*. By contrast, the lower momentum (*p*+, 0)_B_ channel likely propagates through the same initial doorway states before crossing to the 7A_1_ and 8B_2_ states and dissociating into Ph(A) and I.^16^ In their preceding work, Hu et al. could only propose a combined reaction lifetime of 290 ± 90 fs for these two channels, whereas here this signature could be separated into Ph(X) and Ph(A) rise times and given upper limits of 114 ± 6 and 310 ± 60 fs, respectively.

Collectively, our results demonstrate that site-selective ionization induced by XUV pulses is a robust probe for measuring time-resolved neutral photochemistry, including product branching ratios, reaction lifetimes, internal energy deposition, and electronic states.

## Supporting Information Available

Background-subtracted ion images detailing angular isotropy, delay-dependent ion momentum distributions, expanded plots of the individual basis functions used in the global fitting, global fit outcomes for I^3+^ and I^4+^, background-subtracted carbon-containing fragment momentum distributions, CDF fits for the (*p*+, 0)_A_ channels, additional modeled Coulomb curves for the (*p*+, 1+) channels using different iodine and carbon cofragments, and UV/IR delay-dependent momentum distributions paired with recoil-frame covariance calculations (PDF)
