# Supplementary material for: Time-Resolved Probing of the Iodobenzene C-Band Using XUV-Induced Electron Transfer Dynamics
Source: ACS Phys Chem Au. 2024 Aug 10;4(6):620–31. doi: 10.1021/acsphyschemau.4c00036 (PMC11613270; doi:10.1021/acsphyschemau.4c00036)
Supplement: Supplementary file 1 — pg4c00036_si_001.pdf [file pg4c00036_si_001.pdf]

Supporting Information for Time-resolved probing of the iodobenzene C-band using XUV-induced electron transfer dynamics

James Unwin,[†] Weronika O. Razmus,[‡] Felix Allum,[¶,§] James R. Harries,[∥] Yoshiaki Kumagai,[⊥] Kiyonobu Nagaya,[#] Mathew Britton,[¶,§] Mark Brouard,[†] Philip Bucksbaum,[§] Mizuho Fushitani,[@] Ian Gabalski,[§,△] Tatsuo Gejo,[∇] Paul Hockett,[††] Andrew J. Howard,[§,△] Hiroshi Iwayama,[‡‡] Edwin Kukk,[¶¶] Chow-shing Lam,[†] Joseph McManus,[†] Russell S. Minns,[‡] Akinobu Niozu,[§§] Sekito Nishimuro,[∥∥] Johannes Niskanen,[¶¶] Shigeki Owada,[⊥⊥,##] James D. Pickering,[@@] Daniel Rolles,[△△] James Somper,[†] Kiyoshi Ueda,[∇∇∇] Shin-ichi Wada,[§§] Tiffany Walmsley,[†] Joanne L. Woodhouse,[‡] Ruaridh Forbes,[¶] Michael Burt,[*,†] and Emily M. Warne[*,†]

†*Chemistry Research Laboratory, Department of Chemistry, University of Oxford, Oxford OX1 3TA, United Kingdom*

‡*School of Chemistry, University of Southampton, Highfield, Southampton SO17 1BJ, United Kingdom*

¶*Linac Coherent Light Source, SLAC National Accelerator Laboratory, 2575 Sand Hill Road, Menlo Park, CA 94025, USA*

§*PULSE Institute, SLAC National Accelerator Laboratory, 2575 Sand Hill Road, Menlo Park, CA 94025, USA*

∥*National Institutes for Quantum Science and Technology (QST), SPring-8, 1-1-1 Kouto, Sayo, Hyogo 679-5148, Japan*

⊥*Department of Applied Physics, Tokyo University of Agriculture and Technology, Tokyo, Japan*

#*Department of Physics, Kyoto University, Kyoto 606-8502, Japan*

@*Department of Chemistry, Graduate School of Science, Nagoya University, Nagoya, Aichi 464-8602, Japan*

△*Department of Applied Physics, Stanford University, Stanford, CA 94305-4090, USA*

∇*Graduate School of Material Science, University of Hyogo, Kouto 3-2-1, Kamigori-cho, Ako-gun, Hyogo 678-1297, Japan*

††*National Research Council of Canada, 100 Sussex Dr. Ottawa, ON K1A 0R6, Canada*

‡‡*Institute for Molecular Science, Okazaki 444-8585, Japan*

¶¶*Department of Physics and Astronomy, University of Turku, FI-20014 Turku, Finland*

§§*Graduate School of Advanced Science and Engineering, Hiroshima University, Higashi-Hiroshima 739-8526, Japan*

∥∥*Department of Chemistry, School of Science, Tokyo Institute of Technology, 2-12-1 W4-10 Ookayama, Meguro-ku, Tokyo 152-8551, Japan*

⊥⊥*Japan Synchrotron Radiation Research Institute, Kouto 1-1-1 Sayo, Hyogo, Japan*

##*RIKEN SPring-8 Center, Kouto 1-1-1 Sayo, Hyogo, Japan*

@@*School of Chemistry, George Porter Building, University of Leicester, Leicester LE1 7RH, United Kingdom*

△△*J. R. Macdonald Laboratory, Department of Physics, Kansas State University, Manhattan, Kansas 66506, USA*

∇∇∇*Department of Chemistry, Tohoku University, Sendai 980-8578, Japan*

E-mail: michael.burt@chem.ox.ac.uk; chemistry@emilywarne.co.uk

1 Determining temporal pump-probe overlap

No independent measurements to determine the time overlap (t_0) of the two pulses were performed, but an approximate estimate was obtained from the onset of the I^{2+} Coulomb curve. This curve originates from two dissociated fragments being ionised by the probe pulse, and so the earliest point it can appear is the delay at which the pump pulse arrives just before the probe pulse. A short delay just before this point (likely within the 50 fs timing precision of the experiment) would therefore be the point at which the two pulses arrive at the same time. The I^{2+} Coulomb curve was used here as its high-intensity signal enabled an accurate determination of the origin of the curve.

2 XUV pulse energy distribution

Figure S1 shows the full XUV pulse energy distribution recorded throughout the experiment.

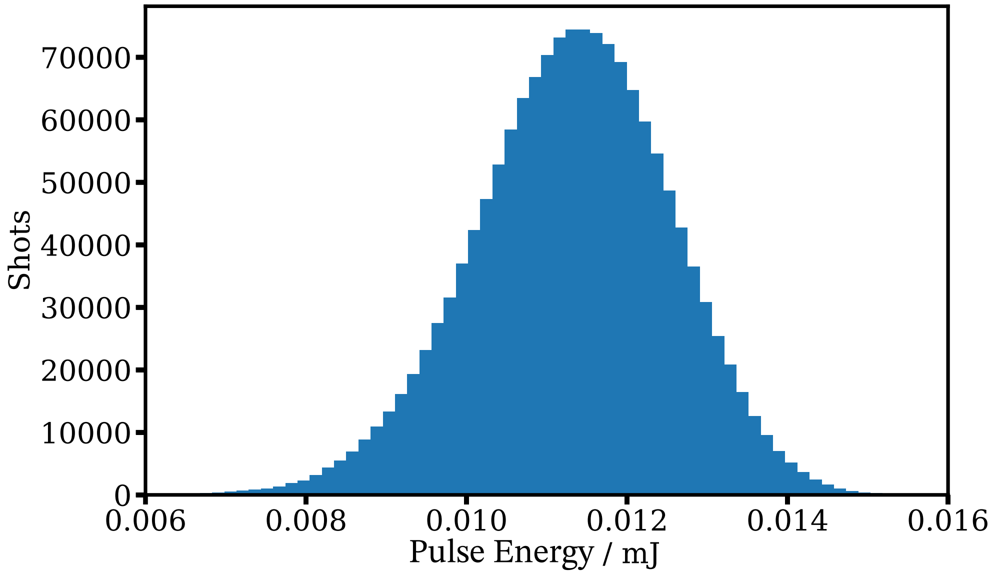

Figure S1: XUV pulse energy distribution recorded throughout the experiment.

3 Iodine cation images

Figure S2 exhibits background-subtracted ion images of $I^{2,3,4,6+}$. The images are summed over all delay ranges where the UV pump pulse precedes the XUV probe pulse. The images are isotropic, consistent with contributions from multiple angular electronic states of different symmetries.

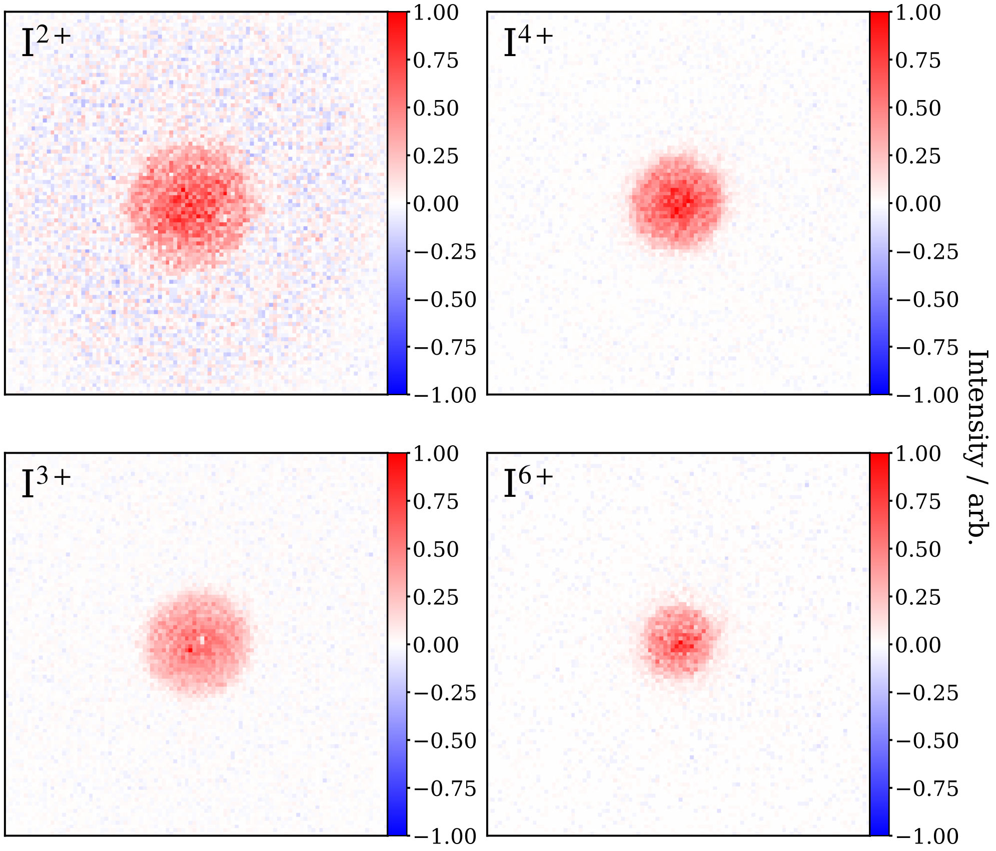

Figure S2: Background-subtracted ion images exhibiting UV-early data. The ion images of each iodine charge state are isotropic.

4 Delay-dependent momentum distributions

Figure S3 demonstrates the delay-dependent momentum distributions arising from the major contributors to the measured mass spectra (Figure 2), prior to any subtraction of pre-t_0 data.

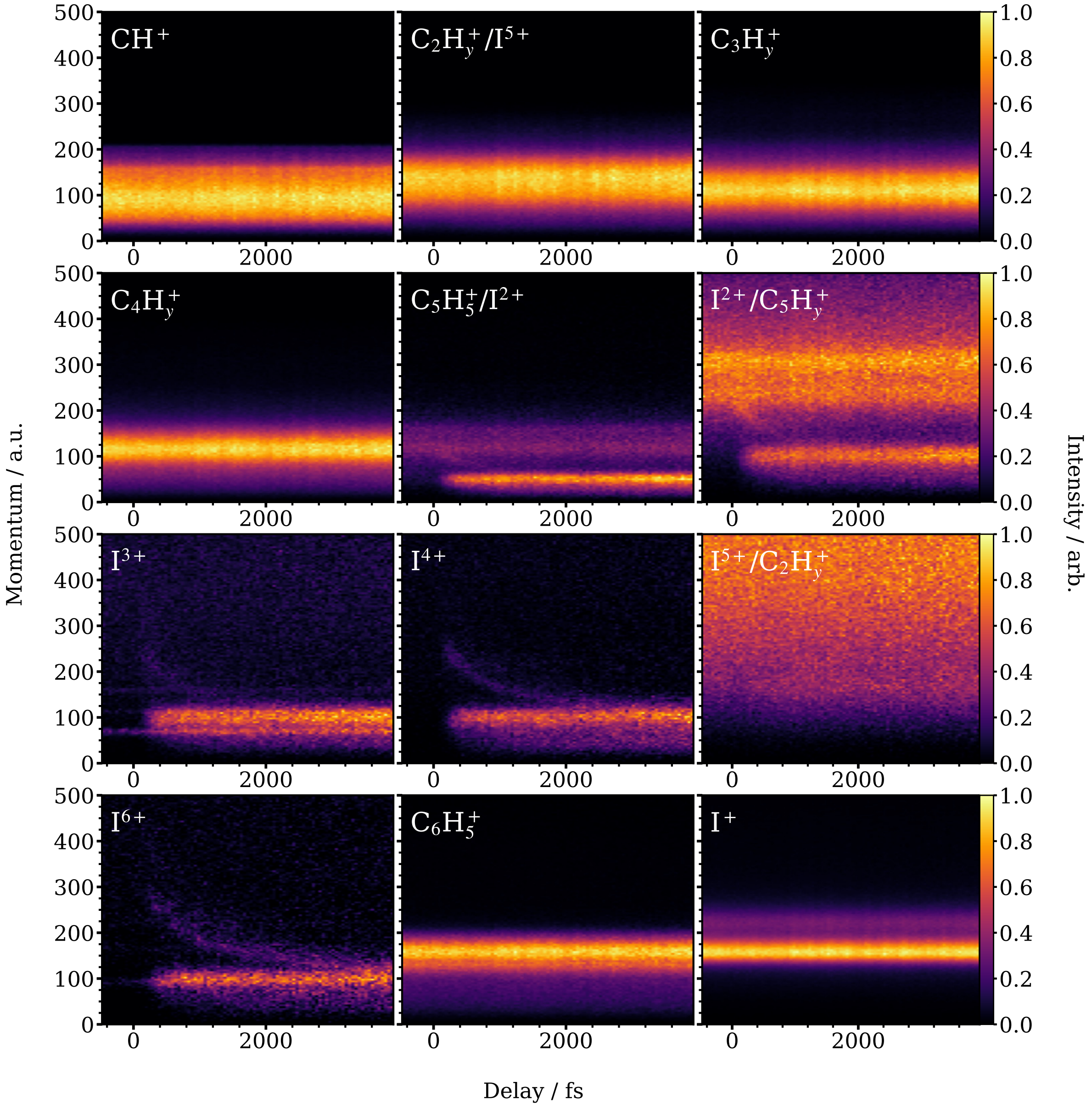

Figure S3: Delay-dependent momentum distributions for all major fragments observed following the fragmentation of iodobenzene. In cases where two labels are shown, the first label indicates the *m*/*z* corresponding to the selected time-of-flight range for that distribution, while the second label indicates that a second *m*/*z* may be present.

5 Basis function determination

Figure S4 demonstrates the basis functions that are used as input for the global fitting method described in the main text. The ground-state depletion, which corresponds to fragmentation of parent molecules by the XUV pulse, was obtained using the pre-t_0 delay data from -500 to -200 fs. The basis function for the high-momentum dissociation was obtained by averaging the data between t_0 and the onset of the low-momentum feature (from 75 fs to 425 fs). This time range was chosen as it covers the initial intensity rises of these channels (for example, see Figure S7), while avoiding any contributions from other features. Finally, the low-momentum function was obtained by subtracting the high-momentum basis function from the late delay data (from 3 to 4 ps). The intensity of each basis function is set to zero at a manually chosen upper momentum limit to prevent any noise in the lower intensity momentum ranges from negatively impacting the quality of the fit, consistent with the work of Razmus *et al.* cited in the main text. This manually chosen upper limit also minimises the effect of the Coulomb curve merging with the channel, as across the selected delay range the Coulomb curve has not reached its asymptotic velocity, and so its presence can skew the Gaussian fits to the basis functions in the main manuscript to higher momenta, due to a positive intensity contribution from the Coulomb curve. Choosing a cutoff point that corresponds to the maximum momentum of the $(p+, 0)_A$ or $(p+, 0)_B$ channels minimises this effect. The effect is most noticeable in the I^{2+} basis function, due to the curves merging with the channel at shorter delay values. Removing the Coulomb curve is less necessary in the high-momentum $(p+, 0)_A$ basis function (particularly in the higher iodine charge states), as the delay range it is generated from occurs prior to the Coulomb curve merging with the channel. Adding a maximum channel momentum cutoff helps minimise the effect of the Coulomb curve on the extracted basis functions, but does not address any increase in intensity within the $(p+, 0)_B$ channel at late delay regions that arise from the curve merging with the channel. This was accounted for by scaling the intensity of the $(p+, 0)_B$ signal by $\sim$13% to account for the low momentum $(p+, 0)_B$ Coulomb curve merging with the channel at late delays, to ensure the basis function represents the contribution of the $(p+, 0)_B$ channel only. This value was determined by calculating the average increase in integrated

intensity between 2-2.5 ps and 3.5-4 ps of a slice of the low momentum channel, to allow isolation of regions prior to, and after, the merging of the Coulomb curve with the $(p+, 0)_B$ channel.

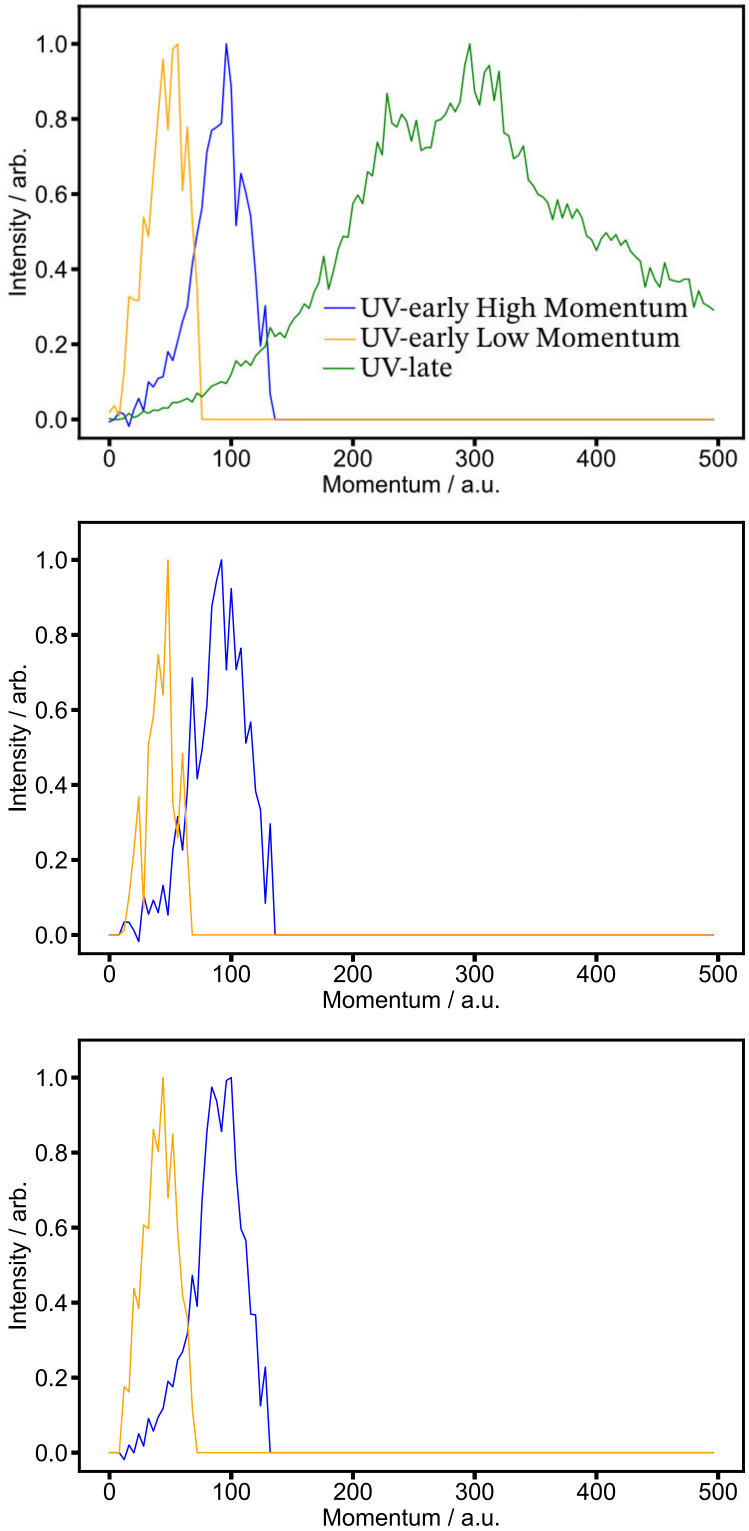

Figure S4: Basis functions extracted for the (p+, 0) enhancements and ground-state depletion channels observed in the I^{2-4+} momentum distributions.

6 Global fit outcomes for I^{3+} and I^{4+}

Figures S5 and S6 illustrate (a) the reconstructed momentum distributions of I^{3+} and I^{4+}, (b) their residual momentum distributions, and (c) the individual contributions of the corresponding basis functions in each case.

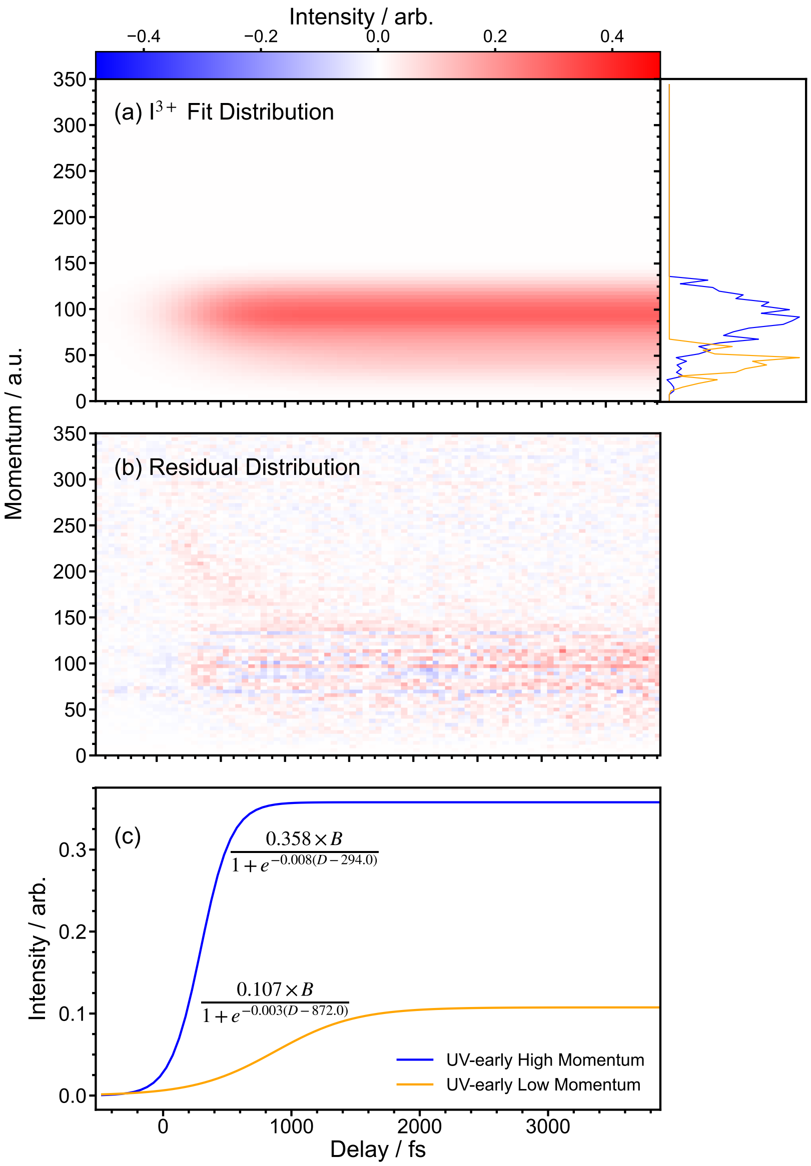

Figure S5: The basis function reconstruction of the I^{3+} pump-probe data, created by fitting eq 1 using a least-squares approach. Panel (a) shows the summed contributions of the momentum-dependent basis functions with respect to delay. The side panel additionally exhibits the selected basis functions used for the global fit, normalised to their maximum intensities. Panel (b) demonstrates the residual data created when the optimised fit is subtracted from the experimental data. Panel (c) shows the individual contribution of each basis function to the reconstructed data along with the optimised parameters determined from the fitting procedure.

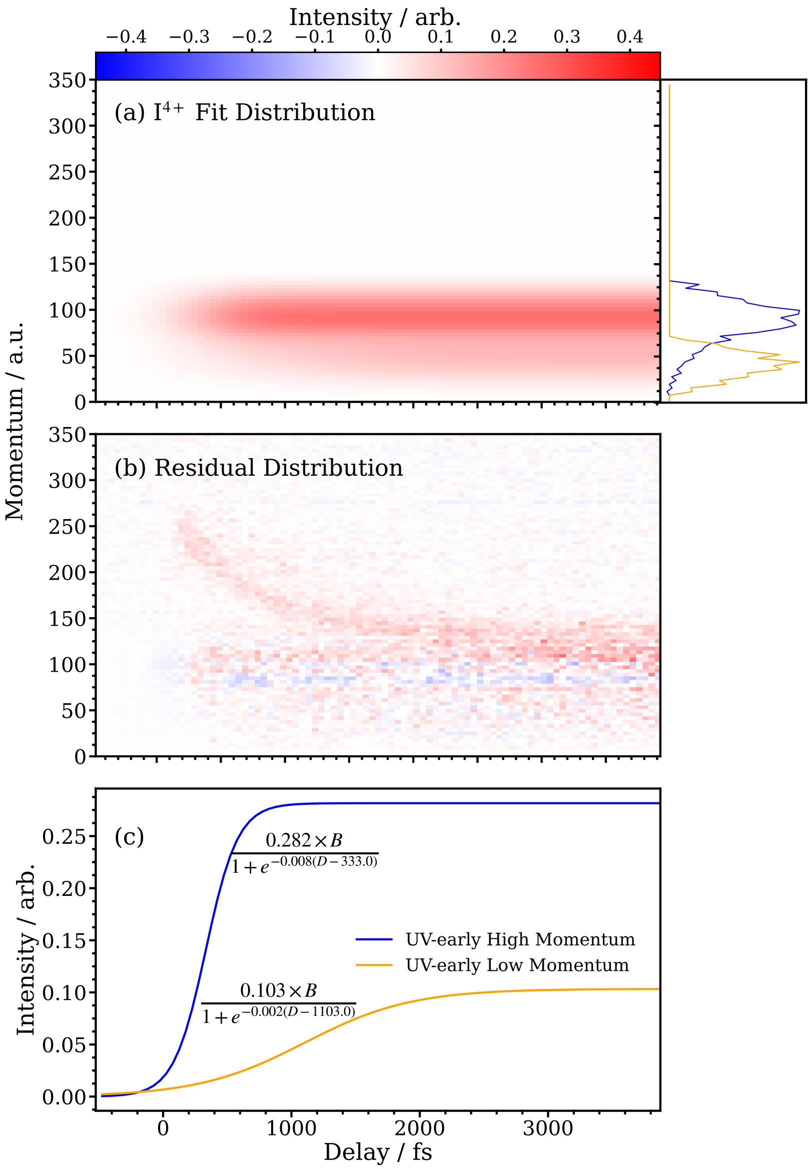

Figure S6: The basis function reconstruction of the I^{4+} pump-probe data (a), prepared the same way as in Fig. S5. The residual momentum distribution (b) and optimised basis set contributions (c) are also shown.

7 CDF fitting of the $(p+, 0)_A$ channels

Figure S7 shows the cumulative distribution function (CDF) fitting of the I^{2-4+} $(p+, 0)_A$ channels, which yield the corresponding channel onset times.

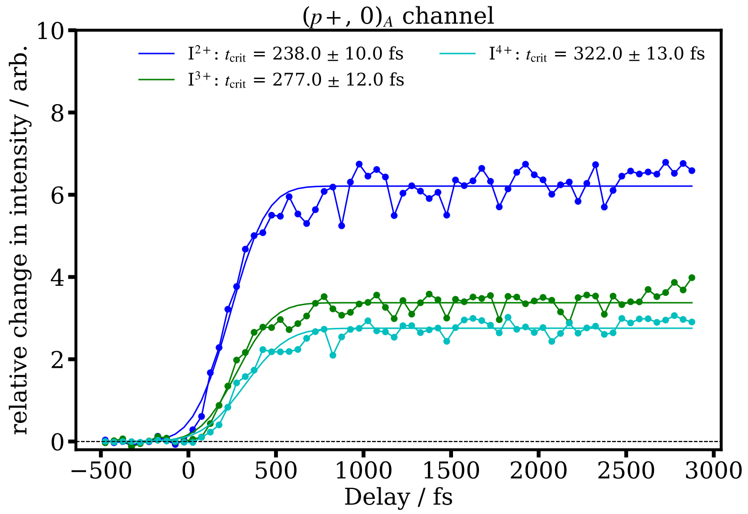

Figure S7: Determination of the onset times for the I^{2-4+} $(p+, 0)_A$ channels using CDF fitting. These onset times correspond to t_{crit} values, which indicate the reaction times at which electron transfer can no longer occur.

8 Time-dependent behaviour of the carbon-containing fragments

Figure S8 provides the background-subtracted and delay-dependent momentum distributions for the *m*/*z* signals corresponding to Ph^+, as well as $C_4H_y^+$, $C_3H_y^+$, and CH_y^+. Subtraction of the ground-state background was accomplished in the same manner as discussed in the main text.

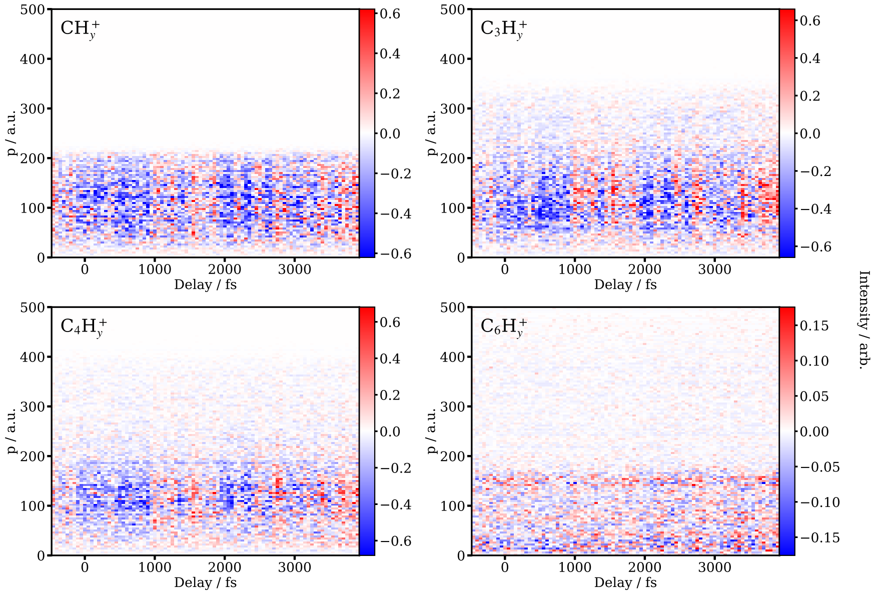

Figure S8: Background-subtracted momentum distributions for carbon-containing fragments that have no overlap with any I^{p+} signals.

9 Coulomb curve modelling of the (p+, 1+) channels

Figure S9 shows the modelled Coulomb curves for $I^{3,4+}$. These were produced in the same manner as discussed in the main text for I^{2+}, using the final velocities of the (p+, 0) neutral dissociation channels, and applying a Coulombic component for each delay value. Strong agreement is seen between the experimental and modelled curves in both cases, supporting the assignment of $C_6H_5^+$ as the cofragment after dissociation.

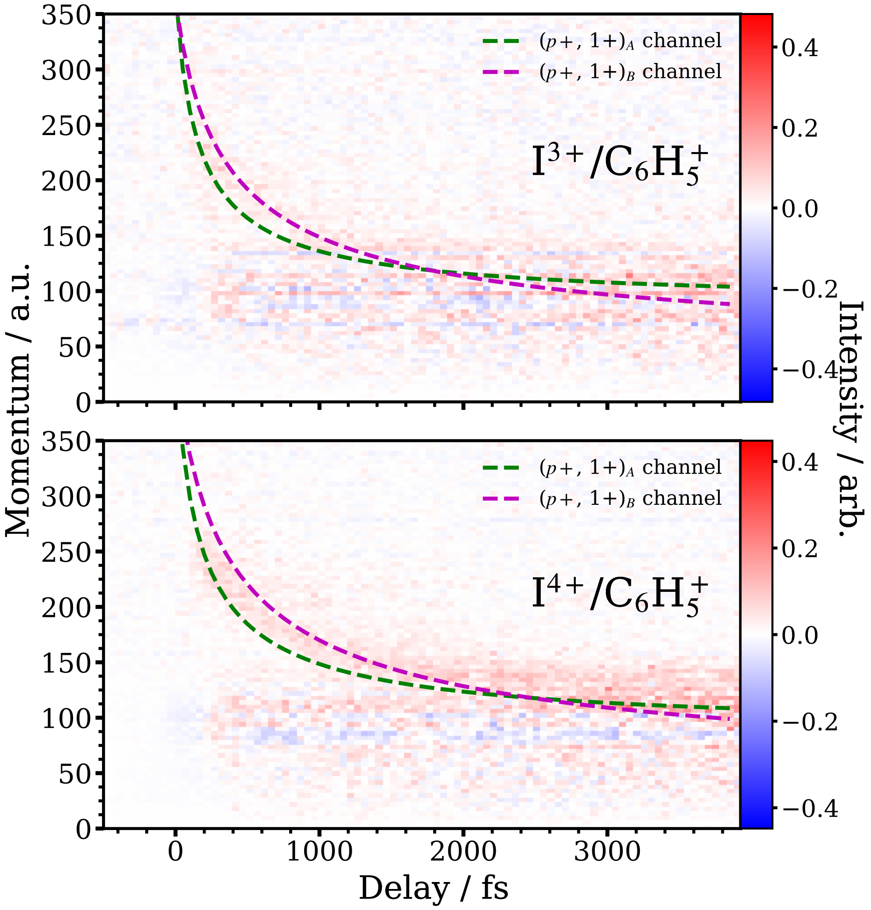

Figure S9: Simulated Coulomb curves for the two-body explosion of $I^{3,4+}$ against Ph^+ at different pump-probe delays, compared with the residual (p+, 1+) curves for I^{3+} and I^{4+} isolated by the global fitting procedure. The $(p+, 1+)_A$ curves were modelled using the fragment velocities produced via the higher momentum $(p+, 0)_A$ dissociation, while the $(p+, 1+)_B$ curve were produced using the lower momentum $(p+, 0)_B$ dissociation.

Figure S10 shows two additional Coulomb curve models, which were produced using different carbon-containing fragments (C_3H_3, and C_2H_2) to those presented in the main text. Both are poor matches to the experimental data. For the purposes of this modelling, it was assumed that the initially dissociated phenyl fragment breaks apart after ionisation into smaller carbon fragments, with no additional momentum imparted to those fragments. Figure S11 illustrates the effect of changing the location of the charge on the phenyl ring on the modelled Coulomb curves. It can be seen that the agreement with the experimental measurements does not significantly change when the charge is shifted further away from the site of C-I cleavage.

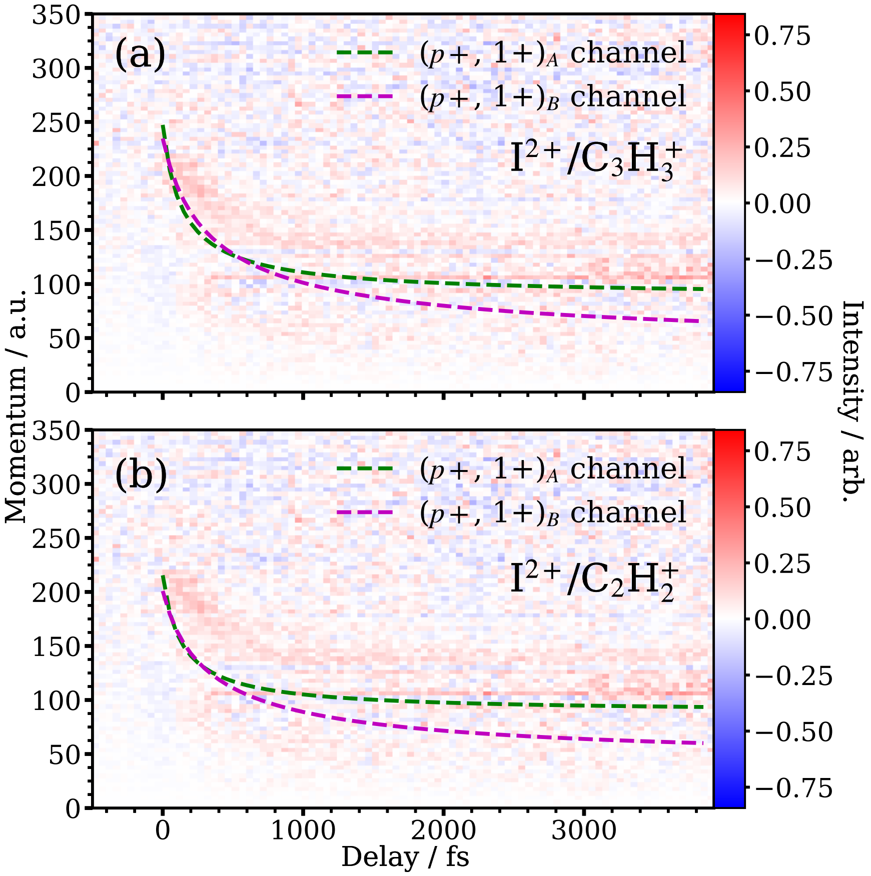

Figure S10: Simulated Coulomb curves for the two-body explosion of $I^{3,4+}$ against $C_3H_3{}^+$ or $C_2H_2{}^+$ at different pump-probe delays, compared with the residual (p+, 1+) curve for I^{2+} isolated by the global fitting procedure.

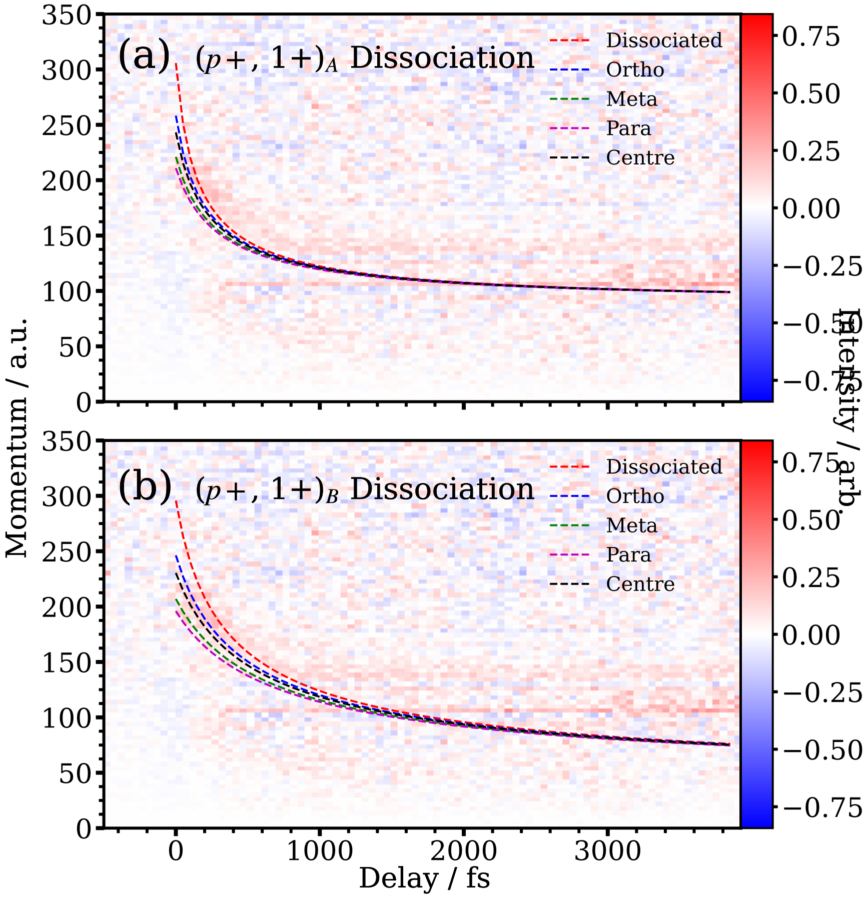

Figure S11: Effect of varying the location of the charge on the phenyl ring for the Coulomb explosion of $I^{3,4+}$ against Ph^{+}. Here, 'dissociated' corresponds to the charge being placed on the carbon involved in C-I cleavage; 'centre' corresponds to the centre of the phenyl ring; and 'ortho', 'meta', and 'para' correspond to the ortho-, meta- and para-positions on the phenyl ring.

10 UV-pump/IR-probe measurements

Figure S12 shows the measured and background-subtracted momentum distributions for I^+ and $C_6H_n^+$ recorded using the UV/IR laser scheme detailed in the main text. The ground-state contributions were removed by subtracting an average of data from 900-300 fs prior to t_0. The presence of time-dependence in the phenyl fragment when using an 800 nm laser supports the assignment of phenyl as a primary neutral cofragment following C-I photolysis at 200 nm.

Using the above data, recoil-frame covariance images (Figure S13) were produced, with both I^+ and $C_6H_n^+$ as the reference ions, for three delay ranges to confirm that the observed time-dependent features in Figure S12 are from the same reaction channel. The delay values were selected to account for when the UV pulse arrived after the IR pulse (t = -0.6 ps), when the UV pulse has just arrived (corresponding to 0.4 ps after t_0) and when the UV pulse arrives several picoseconds before the IR (corresponding to 3.5 ps before t_0).

The covariance was determined for each delay range, and the UV-late covariance was then subtracted from the other two delays. The presence of covariant back-to-back recoil after subtraction confirms that the time-dependent features are from the same reaction channel. The lower radii of these features at later pump-probe delays is explained by the reduced Coulombic repulsion at greater interfragment separations, which results in lower fragment momenta.

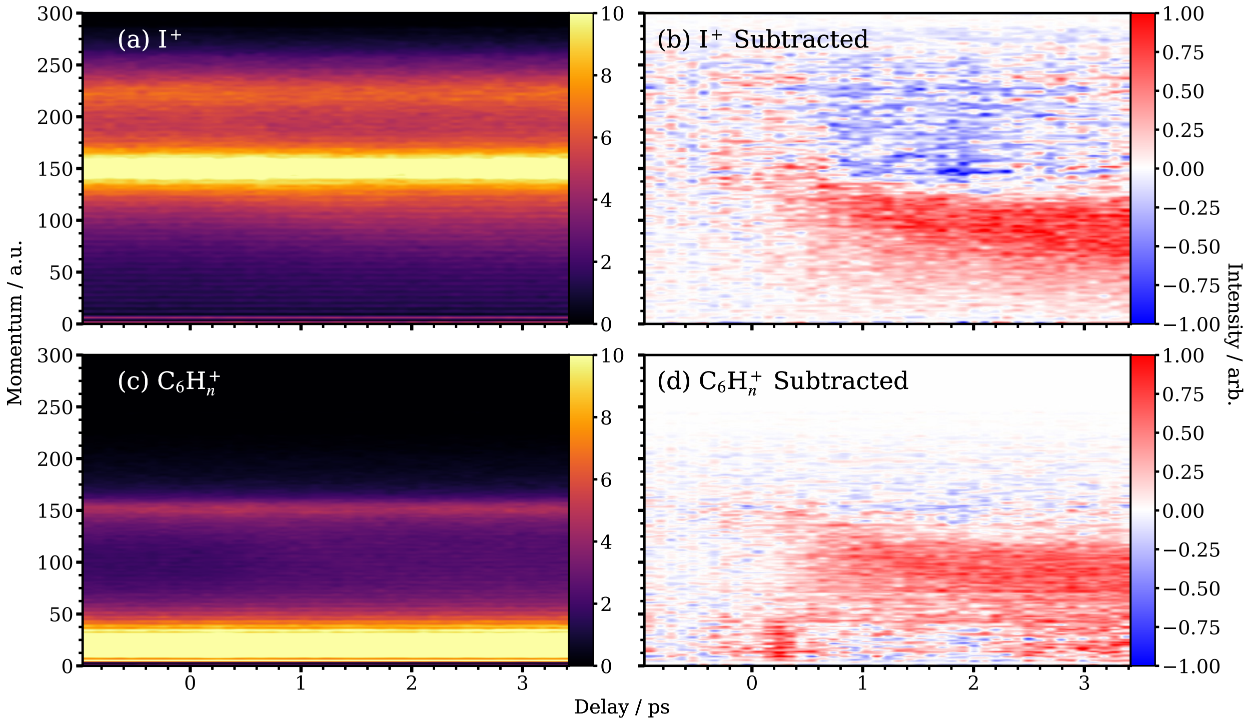

Figure S12: Delay-dependent and background-subtracted momentum distributions for I$^+$ (panels (a) and (b)) and Ph$^+$ (panels (c) and (d)), measured using a UV/IR pump-probe scheme.

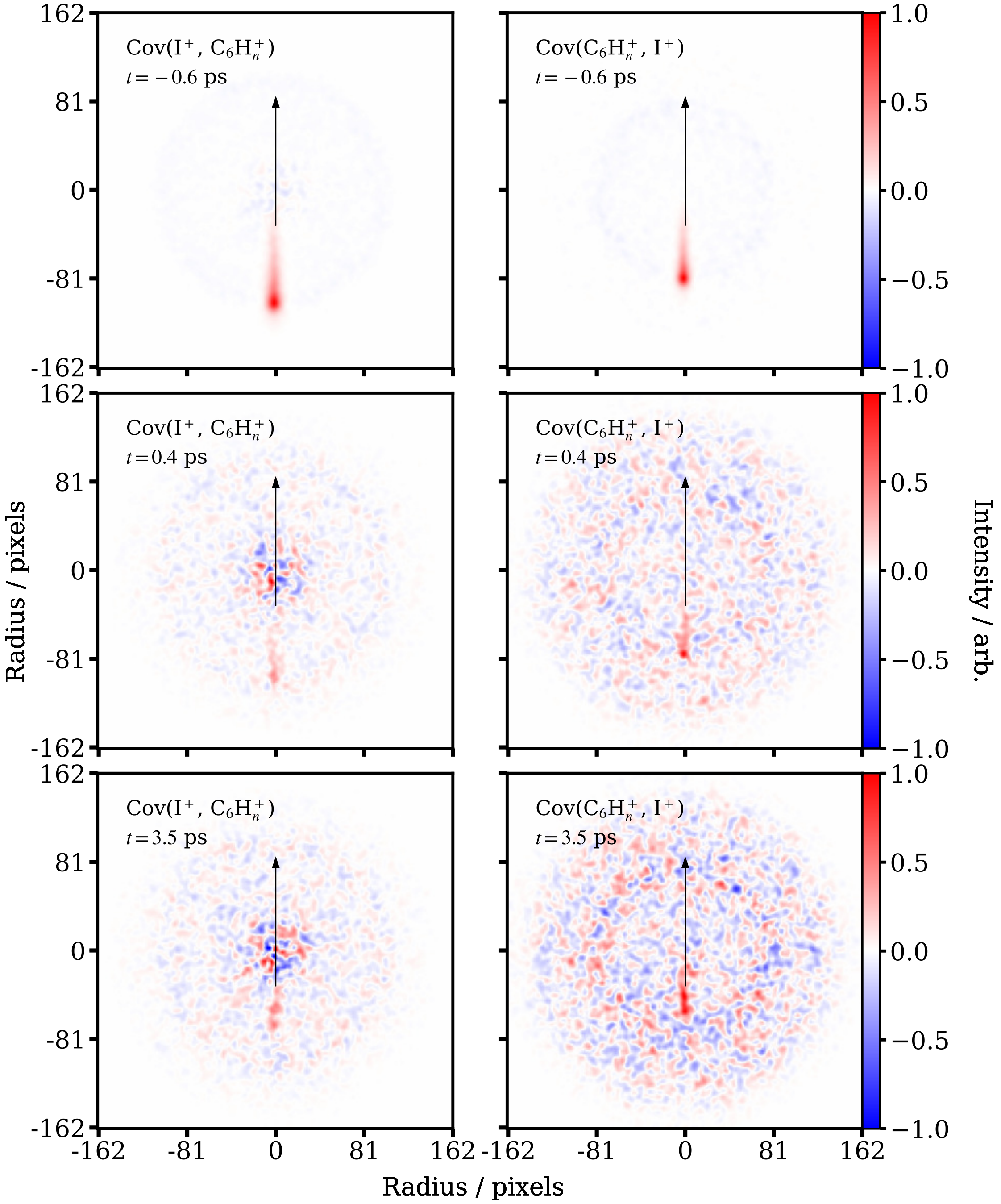

Figure S13: Recoil-frame covariance images for I^+ with $C_6H_n^+$. Three time regions are shown: when the UV pulse arrives after the IR ($t < 0$) ps, when the UV pulse arrives 0.4 ps before the IR, which corresponds to when there is a strong Coulombic repulsion between fragments, and when the UV pulse arrives 3.5 ps before the IR and there is minimal Coulombic repulsion. The intensity is independently normalised for each image. The black arrows correspond to 0° recoil angles.
